# Evaluating the performance of multi-omics integration: a thyroid toxicity case study

**DOI:** 10.1007/s00204-024-03876-2

**Published:** 2024-10-23

**Authors:** Sebastian Canzler, Kristin Schubert, Ulrike E. Rolle-Kampczyk, Zhipeng Wang, Stephan Schreiber, Hervé Seitz, Sophie Mockly, Hennicke Kamp, Volker Haake, Maike Huisinga, Martin von Bergen, Roland Buesen, Jörg Hackermüller

**Affiliations:** 1 Helmholtz Centre for Environmental Research, UFZ, 04318 Leipzig, Germany; 2 BASF Metabolome Solutions GmbH, 10589 Berlin, Germany; 3 Experimental Toxicology and Ecology, BASF SE, 67056 Ludwigshafen, Germany; 4 Institut de Génétique Humaine UMR 9002 CNRS-Université de Montpellier, 34396 Montpellier Cedex 5, France

**Keywords:** Multi-omics, Toxicology, Chemical exposure, Risk assessment, Data integration

## Abstract

Multi-omics data integration has been repeatedly discussed as the way forward to more comprehensively cover the molecular responses of cells or organisms to chemical exposure in systems toxicology and regulatory risk assessment. In Canzler et al. (Arch Toxicol 94(2):371–388. 10.1007/s00204-020-02656-y), we reviewed the state of the art in applying multi-omics approaches in toxicological research and chemical risk assessment. We developed best practices for the experimental design of multi-omics studies, omics data acquisition, and subsequent omics data integration. We found that multi-omics data sets for toxicological research questions were generally rare, with no data sets comprising more than two omics layers adhering to these best practices. Due to these limitations, we could not fully assess the benefits of different data integration approaches or quantitatively evaluate the contribution of various omics layers for toxicological research questions. Here, we report on a multi-omics study on thyroid toxicity that we conducted in compliance with these best practices. We induced direct and indirect thyroid toxicity through Propylthiouracil (PTU) and Phenytoin, respectively, in a 28-day plus 14-day recovery oral rat toxicity study. We collected clinical and histopathological data and six omics layers, including the long and short transcriptome, proteome, phosphoproteome, and metabolome from plasma, thyroid, and liver. We demonstrate that the multi-omics approach is superior to single-omics in detecting responses at the regulatory pathway level. We also show how combining omics data with clinical and histopathological parameters facilitates the interpretation of the data. Furthermore, we illustrate how multi-omics integration can hint at the involvement of non-coding RNAs in post-transcriptional regulation. Also, we show that multi-omics facilitates grouping, and we assess how much information individual and combinations of omics layers contribute to this approach.

**Supplementary Information:**

The online version contains supplementary material available at 10.1007/s00204-024-03876-2.

## Introduction

When exposed to hazardous chemicals, a cell or organism triggers a series of events at the molecular level. These events involve a diverse set of biomolecules with different physicochemical properties. Single-omics methods are limited to identifying only certain types of biomolecules. Therefore, multi-omics or cross-omics approaches are necessary to capture the complete molecular response to a toxicant (Dellafiora and Dall’Asta 2017; Escher et al. 2017; Schwartz 2020; Amorim et al. 2023; Abdelkader et al. 2023).

In Canzler et al. (2020), we reviewed the state of the art in applying multi-omics approaches in toxicological research and chemical risk assessment. We formulated best practices for the experimental design of multi-omics studies, omics data acquisition, and subsequent omics data integration and discussed a regulatory application. Our review revealed that multi-omics data sets for toxicological research questions were rare. We could not identify any such data set for more than two omics layers that adhered to our derived best practices.

The reviewed data sets lacked paired-sample designs, which was a momentous restriction. As discussed in the review, the most powerful data integration approaches involve simultaneous rather than sequential integration. Sequential integration is based on analyzing each single-omics layer separately, for instance, regarding pathway enrichment, and subsequently combining them. In contrast, simultaneous integration aims to analyze all omics layers jointly by identifying shared sources of variance across them. However, simultaneous integration depends on a paired-sample design, which means that the samples in each omics layer must have an inherent and consistent ordering, such as being from the same sequence of animals or cell culture batches.

Due to these restrictions in data availability at the time of writing (Canzler et al. 2020), we could not fully evaluate the power of simultaneous integration approaches. Also, we could only discuss the potential of including or excluding specific omics layers without the option to compute the specific contribution of individual omics layers to detecting a pathway response.

The thyroid is a vital endocrine gland and its hormones 3,3,5-Triiodo-L-thyronine (T3) and L-thyroxine (T4) regulate a number of physiological processes, including cellular growth, embryonic development, differentiation, metabolism, and proliferation (Cheng et al. 2010). The thyroid hormone production is initiated by the hypothalamus via release of the thyrotropin-releasing hormone (TRH), which in turn stimulates the secretion of the thyroid stimulating hormone (TSH) from the anterior pituitary gland. When TSH binds its receptor on thyroid follicular cells synthesis and release of thyroid hormones are initiated. The thyroid gland as one of the relevant elements in European endocrine disruption assessments (EU Commission Delegated Regulation of 19th Dec 2022 amending Regulation (EC) No 1272/2008) is under specific considerations. Thyroid toxicity in animals can occur through various mechanisms: (1) Direct thyroid toxicity primarily affects the follicular cells responsible for the synthesis, storage, and secretion of T3 and T4, e.g., through impacts on the thyroid peroxidase (TPO) and the sodium-iodide symporter (NIS) (Cunha and van Ravenzwaay 2005). (2) Indirect thyroid toxicity involves multiple mechanisms, including alterations in thyroid hormone metabolism and clearance. For example, activation of liver nuclear receptors can induce liver enzymes, leading to increased thyroid hormone glucuronidation and excretion (Foster et al. 2021). Additionally, substances interfering with serum transport proteins might also lead to increased thyroid hormone excretion (Melching-Kollmuss et al. 2023).

Propylthiouracil (PTU) is a pharmaceutical used to treat hyperthyroidism in human. It is one of the most often used reference compounds to induce direct thyroid toxicity via the inhibition of TPO leading to thyroid hormone deficiency and increased TSH secretion in rats. In consequence, the pituitary and thyroid glands will eventually show signs of hypertrophy and hyperplasia coupled with brain heterotopia, as well as neurobehavioral changes (Mellert et al. 2003; O’Shaughnessy et al. 2018). Phenytoin, an anticonvulsant used to treat and prevent seizures, triggers indirect thyroid toxicity via upregulation of biotransformation enzymes in the liver that result in increased clearance of thyroid hormones (Kamp et al. 2012).

Here, we report on a multi-omics study on thyroid toxicity that complies with the best practices reported in Canzler et al. (2020). We induced’s direct and indirect thyroid toxicity through PTU and Phenytoin, respectively, in a 28-day oral rat toxicity study that also included a 14-day recovery phase. We collected clinical and histopathological data and six omics layers from the thyroid and liver. We demonstrate that the integration of multiple omics layers is superior to single-omics analysis in detecting key events and gaining mechanistic understanding and that in most cases, simultaneous integration is outperforming a sequential approach. We further demonstrate that multi-omics data facilitate grouping and show how much information individual and combinations of omics layers contributed to this approach. Finally, we discuss the current state of using (multi-) omics data in regulatory decision-making.

## Materials and methods

### Animal experiment and sampling

The oral toxicity study was designed with different lengths of administration periods, i.e., 14 and 28 days of test substance administration and 28 days plus a 14-day recovery period. A graphical overview of the experimental design is shown in Supplementary Fig. S1. Male Wistar rats (strain: RjHan:WI) were purchased at different ages by Janvier Labs (CS 4105 Le Genest-Saint-Isle, 53941 Saint-Berthevin Cedex, France) and free of clinical signs. Age at delivery and acclimatization periods (8–11 weeks) were selected to ensure a common age at sacrifice. The sex of the animals was chosen to ensure enough tissue material to be examined. In addition, the derived results should be comparable to animals of frequently performed study types, i.e., OECD 408 and 422.

The rats were housed in groups of 5 animals in polysulfonate cages (Tecniplast$\circledR$, Hohenpeißenberg, Germany; floor area approx. 2065 cm$^{2}$) with dust-free wooden bedding. Wooden gnawing blocks (Type NGM E-022; Abedd Lab. & Vet. Service GmbH, Vienna, Austria) were provided to the animals for environmental enrichment. The animal studies were performed with the approval of the local regulatory agencies (Nr: 23 177-07/G 19-3-061).

The experiments were performed in accordance with the OECD test guidelines, applying substance concentrations in the diet of 5 and 50 ppm for Propylthiouracil (PTU) and 300 and 2400 ppm for Phenytoin. Concentrations were selected based on previous studies (Mellert et al. 2003; Cunha and van Ravenzwaay 2005; Kamp et al. 2012; Mattes et al. 2014; Montoya et al. 2014). The test substances were administered over a period of 2 and 4 weeks to groups of 10 male rats, and test substance intake was calculated accordingly. Ten control animals for each period received the vehicle only, i.e., maintenance diet. The same holds for the animals in the recovery phase after treatment.

Thyroid-related hormones (T3, T4, and TSH) were evaluated for all animals. Blood samples were taken from fasted animals by puncturing the retrobulbar venous plexus under isoflurane anesthesia. Sampling and examination were carried out in a computer-generated, randomized sequence on study days 14/15 (short exposure), 28/29 (long exposure), and 42/43 (recovery phase).

### Pathology

After sacrifice, a complete necropsy was performed. Liver and thyroid gland weights were recorded. Relative organ weights refer to terminal body weights. Statistical analysis of organ weights was performed using a non-parametric one-way analysis (Kruskal–Wallis-H test, two-sided) and the Wilcoxon-test (two-sided) (Nijenhuis and Wilf 1978; Miller 1981; Hettmansperger 1984). After weighing, tissues were preserved in 4% neutral buffered formaldehyde solution and embedded in paraffin. From the liver and left thyroid gland, sections of 4 $\upmu$m were prepared, stained with hematoxylin and eosin (H &E), and assessed by light microscopy. Three separate samples for omics examinations were stored at – 80$^{\circ }$C from the liver of each animal. From the right thyroid gland (with parathyroid gland), serial sections were prepared with a cryostat, split alternating in three separate samples, and stored at – 80$^{\circ }$C for omics examinations. Liver and thyroid samples for transcriptomics data generation were additionally treated with 1 ml QIAzol (QIAGEN GmbH, Hilden, Germany) to ensure stabilization of RNA molecules.

### Transcriptomics

Thyroid tissue was ground in QIAzol Lysis Reagent (QIAGEN) using MixerMill MM 400 (RETSCH GmbH, Haan, Germany). For liver tissue, the QIAzol supernatant was used after vortexing for grinding. Total RNA was extracted using miRNeasy Mini Kit according to the manufacturer’s instructions (QIAGEN). DNA contamination was removed with Ambion TURBO DNA-free Kit according to the manufacturer’s instructions (Thermo Fisher Scientific Inc., Waltham, MA, USA). RNA was cleaned and concentrated by ethanol precipitation, and concentration was measured with the Qubit 2.0 instrument using the Quant-iT RNA kit (Thermo Fisher Scientific). RNA integrity was checked with Agilent RNA 6000 Nano Assay using Agilent 2100 Bioanalyzer system (Agilent Technologies, Santa Clara, CA, USA).

We selected 5 out of 10 animals from each treatment group for long and short transcriptomics with the best liver RIN values (see Supplementary Fig. S5).

*Long RNA-Seq.* Ribosomal RNAs were removed from 250 ng total RNA using NEBNext rRNA depletion kit for human/mouse/rat according to the manufacturer’s instructions (New England Biolabs GmbH, Frankfurt, Germany). Dual-indexed, strand-specific library for whole transcriptome sequencing was prepared with NEBNext Ultra II Directional RNA Library Prep kit (New England Biolabs GmbH) following the manufacturer’s instructions. Libraries were evaluated and quantified using Agilent 2100 Bioanalyzer with Agilent RNA 6000 Nano Assay (Agilent) and Qubit 2.0 instrument with Quant-iT dsDNA kit (Thermo Fisher Scientific). Equimolar-pooled libraries were size-selected (200-1000 bp) using 2% agarose gel and MinElute Gel Extraction Kit (Qiagen). Paired-end sequencing was performed at the DRESDEN concept Genome Center on a NovaSeq 6000 system (Illumina, San Diego, CA, USA) using XP workflow and 1.5v SBS chemistry with 2 x 100 bp reads, targeting  40 million fragments per sample. Raw data (bcl files) were demultiplexed using bcl2fastq v2.20 (Illumina).

*Short RNA-Seq.* Libraries were prepared by the MGX sequencing facility (Montpellier, France) using the NEXTflex Small RNA-Seq Kit v3 (Bioo Scientific). They were evaluated and quantified by chromatographic analysis (Fragment Analyzer, kit: High sensitivity NGS) and by qPCR (on a Roche Light Cycler 480). Libraries were pooled in an equimolar mix, denatured, diluted to 300 pM, and hybridized on the flow cell. Sequencing was done on a NovaSeq 6000 (Illumina) with the NovaSeq Reagent Kit (single-end, 100 cycles). Image processing used NovaSeq Control Software (Illumina) and base calling used RTA software (Illumina). Fastq files were generated with bcl2fastq v2.20 (Illumina).

*Data deposition.* Demultiplexed fastq files of both experiments were deposited at NCBI SRA with BioProject identifier PRJNA695243.^1^

### Proteomics

*Protein extraction.* Proteins were extracted using a lysis buffer (pH: 7.4), including 150 mM NaCl (ROTH, Germany), 10 mM TRIS–HCl (ROTH, Germany), 0.1% SDS (SERVA, Germany), 1% Triton X-100 (SERVA, Germany), 1% Sodium deoxycholate monohydrate (abcr, Germany), 5 mM EDTA (Sigma-Aldrich, USA), complete protease inhibitor (Roche, Germany), PhosSTOP phosphate inhibitors (Roche, Germany). Frozen samples were milled with steel balls using a TissueLyser II, then centrifuged at 4$^{\circ }$C for 15 s at 12,000 rpm, incubated for 1 h at 4$^{\circ }$C with gentle swirling, and centrifuged again at 4$^{\circ }$C for 15 min at 14,000 rpm. Protein concentrations were determined by DC protein assay kit (BioRad, USA) with slight modifications to the manufacturer’s instructions. All samples were diluted to the same concentration with lysis buffer. A pooled sample from each tissue served as an internal reference sample (IRS) for normalization.

*Proteomics sample preparation.* Sample preparation and measurement followed a previously described method with minor adjustments (Wang et al. 2021). For TMT-based untargeted proteomics, 25 $\upmu$g of protein was processed using the SP3 paramagnetic bead approach (Hughes et al. 2019). Samples were reduced, alkylated, and enzymatically cleaved with trypsin (1:50), omitting the 10% formic acid addition step. Peptides were labeled with 0.125 mg TMTpro reagent (16plex, Thermo Fisher Scientific, USA) in ACN, followed by quenching, combining TMT-labeled samples into mixes, and peptide clean-up. Each TMT mix included samples from different treatment groups, controls, and pooled IRS samples.

*LC-MS/MS measurement.* Two SP3-prepared fractions were analyzed by LC–MS/MS using a Q Exactive HF mass spectrometer (Thermo Fisher Scientific) coupled to an UltiMate 3000 RSLCnano system (Dionex) with a TriVersa NanoMate electrospray ionization source (Advion), as in Wang et al. (2021) with minor modifications. Samples were loaded onto a trapping column (Acclaim PepMap 100 C18, 3 $\upmu$m, 75 $\upmu$m $\times$ 5 cm, Thermo Fisher Scientific) at 5 $\upmu$L/min for 3 min with a mobile phase of 98% water/2% ACN/0.05% trifluoroacetic acid. They were then separated on an analytical column (Acclaim PepMap 100 C18, 3 $\upmu$m, 75 $\upmu$m $\times$ 25 cm) at 300 nL/min using a 180-min gradient. The top 15 precursor ions were isolated and fragmented.

*Database search.* Raw data were searched against the UniprotKB Rattus norvegicus reference proteome (29944 entries, Aug 3rd, 2020) and a contaminant list (246 entries, MaxQuant 1.6.17.0) using Proteome Discoverer (PD, v2.4.1.15, Thermo Scientific). Parameters included carbamidomethylation (C) and TMTpro (K and peptide N-terminus) as fixed modifications, and oxidation (M) and acetylation (protein N-terminus) as dynamic modifications. The precursor mass tolerance was 10 ppm, and the product ion tolerance was 0.02 Da. Trypsin cleavage was assumed, allowing up to two missed cleavages. Total peptide amount-based normalization was applied, requiring a minimum of two peptides per protein. Data files are available on PRIDE (Perez-Riverol et al. 2019) with ID PXD026835.^2^

### Plasma metabolomics

Blood sampling and extraction have been described above. From each animal, 1 ml of blood was collected with 10 $\upmu$L of 10% EDTA as an anticoagulant. Samples were centrifuged, and the plasma was separated under cooling. Samples were covered with an N2 atmosphere to minimize oxidative damage and subsequently stored at – 80$^{\circ }$C (or on dry ice during shipment).

*Metabolite profiling.* MS-based metabolite profiling of blood plasma followed this protocol: 60 $\upmu$L rat plasma was extracted with 1500 $\upmu$L buffer (methanol, dichloromethane, water, toluene, and ammonium acetate) using a ball mill. Internal standards were added, and after centrifugation (12,000 rpm, 10 min, 12$^{\circ }$C), 100 $\upmu$L of the extract was used for LC–MS/MS. For reversed-phase and hydrophilic interaction liquid chromatography, 2.5 $\upmu$L of the extract was injected, followed by MS/MS detection using positive and negative ionization modes. Reverse-phase HPLC and HILIC gradient elution were performed with specific solvent mixtures. Another extract aliquot was phase-separated for GC analysis. The polar and lipid phases were derivatized and analyzed by gas chromatography–mass spectrometry (GC–MS). Steroid hormones, catecholamines, and metabolites were measured by online SPE-LC–MS/MS. All samples were analyzed once in a randomized sequence to avoid analytical shifts, with data normalized to the median of reference samples (ultrapools). In plasma, 495 semiquantitative metabolites were analyzed using the single peak signal and a normalization strategy based on patent WO2007012643A1 (Walk et al. 2011). Details are given in Supplementary Sect. S2.1. Data set is available at Zenodo.^3^

### Tissue metabolomics

We selected the same samples for tissue metabolomics data generation as for the transcriptomics experiments. Approximately 100 mg of wet-weight liver tissue was separated at room temperature. All available thyroid gland material was used due to its small size. The tissue was mixed with 5x extraction solution (50% acetonitrile, 50% water) and homogenized with steel balls and a tissue slicer for 10 min at 30 Hz. After 2 min of centrifugation at 14,000 rpm, the supernatant containing metabolites was collected and stored at – 80$^{\circ }$C for later analysis.

The MxP Quant 500 kit (Biocrates Life Science, Austria) was used to quantify 630 metabolites across 26 biochemical classes, following the manufacturer’s instructions. Sample preparation occurred on a 96-well plate, including seven calibration standard levels and three QC standards (QC1, QC2, QC3), with four additional QC2 samples distributed across the plate. Samples were added to the adsorption pad system, dried under nitrogen for 30 min, then derivatized with 50 $\upmu$L 5% PITC for 60 min, followed by 60-min drying under nitrogen flow. A 300 $\upmu$L extraction solvent (5 mM ammonium acetate in methanol) was added and shaken for 30 min. Samples were eluted by centrifugation for 30 min at 450 rpm, separated into two parts, and diluted for FIA and LC measurement. Measurements were conducted using the QTRAP 5500 system (AB SCIEX) equipped with an LC 1290 Infinity system (Agilent). For FIA, solvent B (290 mL methanol + 1 ampule FIA Mobile Phase Additive) was injected with specific flow rate adjustments. The compound-specific MRM transitions were determined in positive ionization mode. For LC, an MxP Quant 500 Column System was used at 50$^{\circ }$C, with eluents A (2000 mL water + 4 mL formic acid) and B (2000 mL acetonitrile + 4 mL formic acid). Specific MRM transitions were measured in positive and negative ionization modes. Details are given in Supplementary Section S2.2. MetIDQ software was used for quantification and quality assurance according to the manufacturer’s instructions. Data sets are available at Metabolomics Workbench (Sud et al. 2016) with StudyID ST002023.^4^

### Phosphoproteomics

Phosphoproteomics was performed on the same liver samples used for transcriptomics data. We applied the SP3-based proteomics approach, which involved reducing, alkylating, and TMT labeling peptides before phosphopeptide enrichment. To enrich a suitable number of phosphopeptides, 200 $\upmu$g of starting protein was used. Phosphopeptides were enriched using High-Select TiO2 and Fe-NTA Phosphopeptide Enrichment Kits (Thermo Fisher, USA) as described in Großkopf et al. (2021) with minor exceptions. Flowthroughs were saved and analyzed as the complementary proteome.

For LC–MS/MS measurement of enriched phosphopeptides, the same setup as for proteome analysis was used, with adjusted LC gradients. TMT-labeled samples were loaded on a trapping column for 3 min, and then separated on an analytical column followed by a 180-min gradient with stepwise adjustments of the mobile phase and 7.5 min loading column wash in the end. Spectra acquisition parameters were similar to the proteome analysis, with maximum injection time set to 150 ms. The top 15 precursor ions were isolated and fragmented, with a maximum IT of 150 ms. Details are given in Supplementary Sect. S2.3.

*Database search.* The same UniprotKB database and PD version were used as for the proteomics data. The workflow was similar, except phosphorylation (S, T, Y) was specified as a dynamic modification. The IMP-ptmRS node calculated phosphorylation confidences and probabilities, and a node for extracting peptide isoform information was included. Total peptide amount-based normalization was applied, with a minimum of one peptide per protein. Data files are available on PRIDE (Perez-Riverol et al. 2019) with the ID PXD030254.^5^

*Phosphosite intensity extraction.* We used an in-house R script to extract intensities for each phosphosite, accounting for the fact that one peptide isoform can contain multiple phosphosites, and vice versa. IRS-based normalization removed TMT batch effects by dividing the peptide isoform intensity in each sample by that of the IRS sample from the same batch. Only phosphosites with a probability above 75 were considered reliable. Phosphosite intensity was calculated by summing the intensities of all peptide isoforms containing that specific phosphosite.

### Preprocessing of omics data and single-omics analysis

The methods for omics data processing and multi-omics integration are briefly explained here. Detailed steps are available as YAML or Rmarkdown files in our GitLab repository.^6^

*Transcriptomics.* Fastq files were processed in uap for reproducible high-throughput data analysis (Kämpf et al. 2019). Sequencing adapters were trimmed using cutadapt v2.1 (Martin 2011). Quality control was performed with fastqc^7^ v0.11.4 and fastx-toolkit v0.0.14 before and after trimming. Reads were aligned to the rat genome Rnor_6.0 (Ensembl Release 102) using hisat2 v2.2.1 (Kim et al. 2019) and sorted with samtools v1.9 (Li et al. 2009). New transcripts were generated using stringtie v2.1.2 (Kovaka et al. 2019) and merged with stringtie-merge. New transcripts were annotated using cuffcompare v2.2.1 (Trapnell et al. 2010). Read counts for known genes or new transcripts were obtained using htseq-count v0.12.4 (Anders et al. 2015). The uap configuration file with parameter settings is available in the git repository.

Count data for each tissue were transformed into a DESeq2 data object (Love et al. 2014), removing features without counts. A variance-stabilizing transformation was applied as suggested by the authors of the MOFA package (Velten et al. 2022). Thyroid samples showed a batch effect due to varying amounts of parathyroid tissue, identified by the RUVSeq R package (Risso et al. 2014). This effect was removed using the removeBatchEffect() function from limma (Ritchie et al. 2015). For multi-omics data integration, both data sets were filtered to include only genes with a baseMean > 10 and a row variance above the 30th quantile. The final data sets contained 12550 genes for thyroid and 12293 genes for liver. The workflows are described in the git repository in file 01_transcriptomics_processing.Rmd.

*Short transcriptomics.* After sequencing, barcoded $3^{\prime }$ adapters were trimmed using cutadapt v2.1. Please see Supplementary Table S1 for individual adapters. Adapter-trimmed reads were mapped to a manually extended rat pre-miRNA catalog^8^ using Hisat2. Sequences were extended by $\geqslant$ 10 nt on each side of the Drosha cleavage sites to account for potential cleavage inaccuracies. Unmapped reads were trimmed by 1 nt on their $3^{\prime }$ end before mapping them again at the next iteration (therefore identifying miRNA isoforms which underwent addition of up to 5 untemplated nucleotides at their $3^{\prime }$ end). Scripts and data files for the processing of short RNA-Seq data are available online.^9^

Both tissue-specific data sets have been processed individually. Again, we applied a variance-stabilizing transformation to the count data. Similar to the long RNA-Seq thyroid data set, this set also showed batch effects that might be caused by varying amounts of parathyroid tissue within each sample. The liver data set revealed two outlier samples with unknown sources of variation. However, for both sets, we applied the batch correction procedure utilizing the sva and limma packages to account for the first surrogate variable. The preprocessing procedure can be found in the git repository

(01_short_transcriptomics_processing.Rmd).

*Proteomics.* The thyroid data set included 10 TMT batches, each containing one sample per treatment group and the IRS. Batches were mean normalized individually for each protein. We then selected the five samples per treatment group with additional transcriptomics data. After filtering for proteins measured in at least 3 of 5 samples, a variance-stabilizing transformation was applied.

The liver data set had 9 TMT batches due to one failed preparation, with each batch containing one sample per treatment group and the IRS. Batches were mean normalized. Filtering for samples with transcriptomics measurements was unaffected by the missing TMT batch, reducing the data set to 5 samples per treatment group before applying the variance-stabilizing transformation. A batch effect was identified by the sva package (Leek et al. 2012) and corrected with limma. The workflow is detailed in file 01_proteomics_processing.Rmd in our git repository.

*Tissue metabolomics.* Although the Biocrates MxP Quant 500 kit typically does not require transformation, we applied a variance-stabilizing transformation to the tissue metabolomics data for consistent preprocessing before data integration. Thyroid and liver samples were preprocessed separately. Metabolites measured in at least 3 out of 5 samples per treatment group were retained, resulting in 510 metabolites for thyroid and 387 for liver. The workflow is described in our git repository in file

01_Tissue_metabolomics_processing.Rmd.

*Plasma metabolomics.* The plasma metabolomics data set was filtered for those 5 samples per treatment group with additional omics layers. Metabolites present in at least 3 out of 5 samples per group were retained, totaling 479 metabolites. A variance-stabilizing transformation was applied. The workflow is detailed in our git repository (01_Plasma_metabolomics_processing.Rmd).

*Phosphoproteomics.* The phosphoproteomics data set included 70 samples; one sample was lost in each of the 5 recovery-specific groups. Phosphorylation sites measured in at least 3 samples per group were retained, followed by a variance-stabilizing transformation. Details are in 01_phosphoproteomics_processing.Rmd in our git repository.

*Single-omics analysis*: Workflows for each individual single-omics analysis to calculate differentially altered features are also located in the git repository.

### Multi-omics data integration and analysis

Preprocessed omics data sets were used to derive four tissue- and treatment-specific multi-omics models using the MEFISTO-framework from the MOFA2 package (Argelaguet et al. 2018; Velten et al. 2022). Thyroid-specific models contain five omics layers (long and short transcriptomics, proteomics, tissue, and plasma metabolomics) and all nine treatment groups. Liver models also included the phosphoproteomics layer. Clinical and histopathological data (e.g., body weights, organ weights, and severity of hyperplasia) were added as covariates. Each model contained five latent factors. The workflows for building the MEFISTO models are in the git repository under 03_MEFISTO_model_[organ]_[compound].Rmd.

Pathway enrichments were conducted with multiGSEA (Canzler and Hackermüller 2020) and details are given in the git repository

(04_pathway_enrichment_[organ]_[compound].Rmd). MicroRNA-based pathway enrichment was calculated with miRPath-v4.0 (Tastsoglou et al. 2023). The workflow is in the git repository in file 01_[organ]_miRNA_pathway_enrichment.Rmd.

The sample classification procedure was based on the euclidean distance of sample factor loadings across all five latent factors and is described in the files 05_grouping_liver_Phenytoin.Rmd and 05_grouping_thryoid_PTU.Rmd.

## Results

### Assessing direct and indirect thyroid toxicity in an oral rat toxicity study

We conducted an oral rat toxicity study to generate biomaterial samples for multi-omics analysis, adhering to the best practices that we had derived in Canzler et al. (2020). Male Wistar rats received either no treatment (control group), low- or high-dose PTU (5 and 50 ppm) or Phenytoin (300 and 2400 ppm) for 14 or 28 days, followed by a 14-day recovery period without treatment.

*Clinical and histopathological results.* In the clinical studies, we observed the expected treatment-related effects, i.e., impaired food intake and body weight development due to treatment with high-dose PTU and Phenytoin. During the recovery phase, animals gained weight again in both treatment groups. No significant clinical effects (food intake, body weight) could be determined for both test chemicals when administered at low concentrations (see Supplementary Sect. S1.2 and Supplementary Figure S2 for details).

**Fig. 1:**
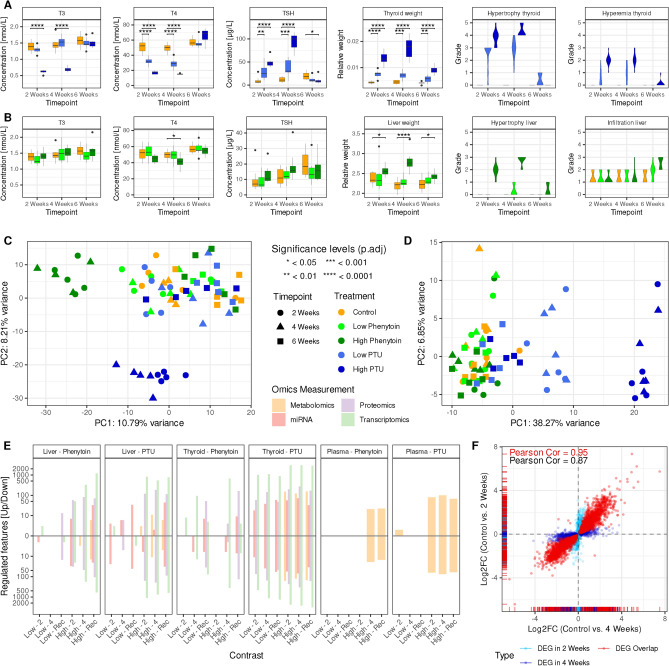
Clinical and histopathological readouts and single-omics data analysis of thyroid and liver samples. **A** Serum thyroid hormone levels, thyroid weight (relative to body weight), and histopathological findings in the thyroid for PTU-treated samples. The severity of hypertrophy and hyperemia is graded from 0 to 5, with 5 being the most severe. Ten animals per treatment group were summarized. Boxes and violins in all facets are grouped in the following order: controls, low-dose, and high-dose PTU. **B** Serum thyroid hormone levels, liver weight (relative to body weight), and histopathological findings in the liver for Phenytoin-treated samples. The severity is graded from 0 to 5, with 5 being the most severe. Ten animals per treatment group were summarized. Boxes and violins in all facets are grouped in the following order: controls, low-dose, and high-dose Phenytoin. **C** Proteomics PCA of all 75 liver samples. **D** Transcriptomics PCA of all 75 thyroid samples. **E** Summary of single-omics data analysis concerning tissue and treatment. Significantly altered features in the transcriptomics, proteomics, tissue metabolomics, and short RNA-Seq data are shown for liver and thyroid samples, while plasma metabolomics results are shown for plasma. The FDR threshold was set to 0.01. For simplicity, only a subset of contrasts is shown: low- and high-dose treatments of 2 and 4 weeks against their respective controls and recovery samples against their respective 4 week treatment. Phosphoproteomics yielded no differentially altered phosphorylation sites in the shown contrasts and hence was not considered here. **F** Comparison of differentially expressed genes of high-dose PTU treatment for 2 and 4 weeks in the thyroid samples. Log2 fold changes of both contrasts are plotted against each other for those genes that were differentially expressed in one (light and dark blue) or both contrasts (red). The Pearson correlation of log2 fold changes was calculated for DEGs found in both contrasts (red) and DEGs exclusively found in one contrast (black)

In the high-dose PTU group, T3 and T4 hormone levels significantly decreased after 2 and 4 weeks of administration, while TSH levels significantly increased (as shown in dark blue in Fig. 1A). Conversely, in the low-dose PTU group, only T4 and TSH levels responded similarly, with more pronounced alterations after 4 weeks (indicated in light blue). After a 2-week recovery phase, all hormone levels in both groups returned to levels comparable to those of the control group, except for a slight but significant decrease in TSH levels in the high-dose PTU group.

The relative weights of thyroid glands showed a significant increase after 2 and 4 weeks of both low-dose and high-dose PTU treatments. After 2 weeks of recovery, animals still showed a significantly increased relative thyroid gland weight, although not as pronounced as before.

PTU-treated animals showed diffuse follicular hypertrophy/hyperplasia (2- and 4-week treatment) with a dose-related increased severity or diffuse hyperplasia (recovery group), respectively. Follicular cell hypertrophy and hyperplasia are characterized by increased size and number of thyrocytes. Cells pile up into the follicular lumen, and the lumen becomes smaller, containing reduced amounts of colloid (Brändli-Baiocco et al. 2018). The hyperplasia in recovery animals was characterized by an increased number of low cuboidal follicular cells. Large, colloid-filled follicles were lined by numerous smaller follicles containing less colloid. This picture corresponded to a decrease in hypertrophy, while hyperplasia was still present. Macroscopically, thyroid glands were enlarged and showed a dark red discoloration. The latter correlated microscopically to a marked hyperemia (Fig. 1A). Macroscopic and histopathologic findings were associated with increased absolute and relative thyroid gland weights (Fig. 1A and Supplementary Fig. S3).

Phenytoin-treated rats exhibited a weak but significant decrease in T4 levels in the high-dose group after 4 weeks of treatment. The mean TSH level in this test group increased by 54% compared to controls without statistical significance. After a 2-week administration period, no significant changes of T3, T4, and TSH serum levels were observed in low and high dose (Fig. 1B, light and dark green colors). After the 2-week recovery phase, all hormone levels were comparable to the controls.

Relative liver weights were significantly increased after 2 and 4 weeks of high-dose Phenytoin treatment. After the 2-week recovery period, animals still showed a significantly but less pronounced increased relative liver weight (Fig. 1B).

After 2- and 4-week treatment, Phenytoin-treated animals of the high-dose groups showed minimal-to-moderate centrilobular hepatocellular hypertrophy; the hypertrophy was accompanied by a centrilobular to intermediate zone fatty change. The centrilobular hypertrophy is characterized by an increased size of hepatocytes around central veins (Thoolen et al. 2010). It is considered to be a correlate for a microsomal enzyme induction in the liver that also may be responsible for the minimal thyroid gland hypertrophy/hyperplasia of follicular cells due to an accelerated degradation of T4 (by induced UDP-glucuronyl transferase activity) which is compensated with increased TSH levels (Papineni et al. 2015). Histopathological findings were associated with significantly increased liver weights (Fig. 1B). We detected minimal centrilobular hypertrophy in the low-dose group after 4 weeks of treatment and in the high-dose group after the recovery phase. Animals of the latter group presented, in addition, an increased severity of lymphoid liver cell infiltrates. However, the significance of these infiltrates is questionable, since this is a frequent background finding in Wistar rats (McInnes 2012).

*Single-omics results.* Using long and short transcriptomics, proteomics, and tissue metabolomics, we generated omics data sets from thyroid and liver tissue samples. Additionally, we performed phosphoproteomics in liver tissue samples and metabolomics measurements in plasma.

Principal component analyses (PCA) for each omics layer and tissue exhibited that the ability to discriminate treatment groups differed between individual omics layers (Supplemental Figures S6 and S7). As an example, the PCA of liver proteomics samples (Fig. 1C) showed a separation of high-dose Phenytoin (dark green) and high-dose PTU-treated samples (dark blue) from the remaining samples, including low-dose treated samples, recovery samples, and controls. Also, the first two principal components did not sufficiently separate samples with different treatment durations (2 versus 4 weeks). The PCA of transcriptomics in thyroid samples, in contrast, displayed a concentration-based separation of low- (light blue) and high-dose PTU samples (dark blue) but did not separate according to the duration of treatment (Fig. 1D).

The analysis of PCAs across all omics layers revealed that low-dose Phenytoin treatment and the duration of administration had only minor effects at the molecular level. Analogously, low-dose Phenytoin resulted in a low number of significantly regulated features across different omics layers in thyroid, liver, and plasma samples (Fig. 1E). For high-dose Phenytoin, on the other side, we detected a pronounced molecular response, especially in the liver, across all five acquired omics layers. Low-dose PTU showed marginal effects in the liver, no effect in plasma metabolomics, and pronounced effects in thyroid samples in proteomics and both short and long RNA-Seq data. High-dose PTU displayed severe effects in thyroid samples in all omics layers and less pronounced effects in the liver and plasma samples.

In the treatment groups and tissues where we observed a molecular response after 4 weeks of treatment, we identified a comparable number of regulated features after 2 weeks of recovery (Fig. 1E). For example, the numbers of regulated features in response to low- and high-dose PTU treatment after 4 weeks versus control in the plot (labeled Low-4 and High-4, respectively) were comparable to those of recovery versus the corresponding 4 weeks treated samples (labeled Low-Rec and High-Rec, respectively) across all omics layers. In addition, we observed a high correlation between 2 weeks treated versus control and four-weeks treated versus control log2 fold changes of those features (Fig. 1F). Genes differentially expressed in thyroid samples after 2 and 4 weeks of high-dose PTU treatment showed almost identical log2 fold changes with a Pearson correlation coefficient of 0.96.

In summary, the single-omics analyses met our expectations. However, we anticipated more pronounced effects from low-dose Phenytoin, where we did not find any discernible differences from controls, and the treatment duration, where 2 and 4 week administration often resulted in comparable outcomes. Furthermore, phosphoproteomics data were not able to distinguish between experimental conditions (Supplemental Figure S7E), and no differentially regulated phosphorylation sites were detected. Therefore, we did not pursue further single-omics analysis of the phosphoproteomics data, but we retained it as an input layer for multi-omics integration, as it could still complement other omics layers. Based on the clinical readouts and single-omics results, we selected two conditions for detailed multi-omics analyses: thyroid samples treated with PTU and liver samples treated with Phenytoin.

### Multi-omics integration with clinical readouts

**Fig. 2:**
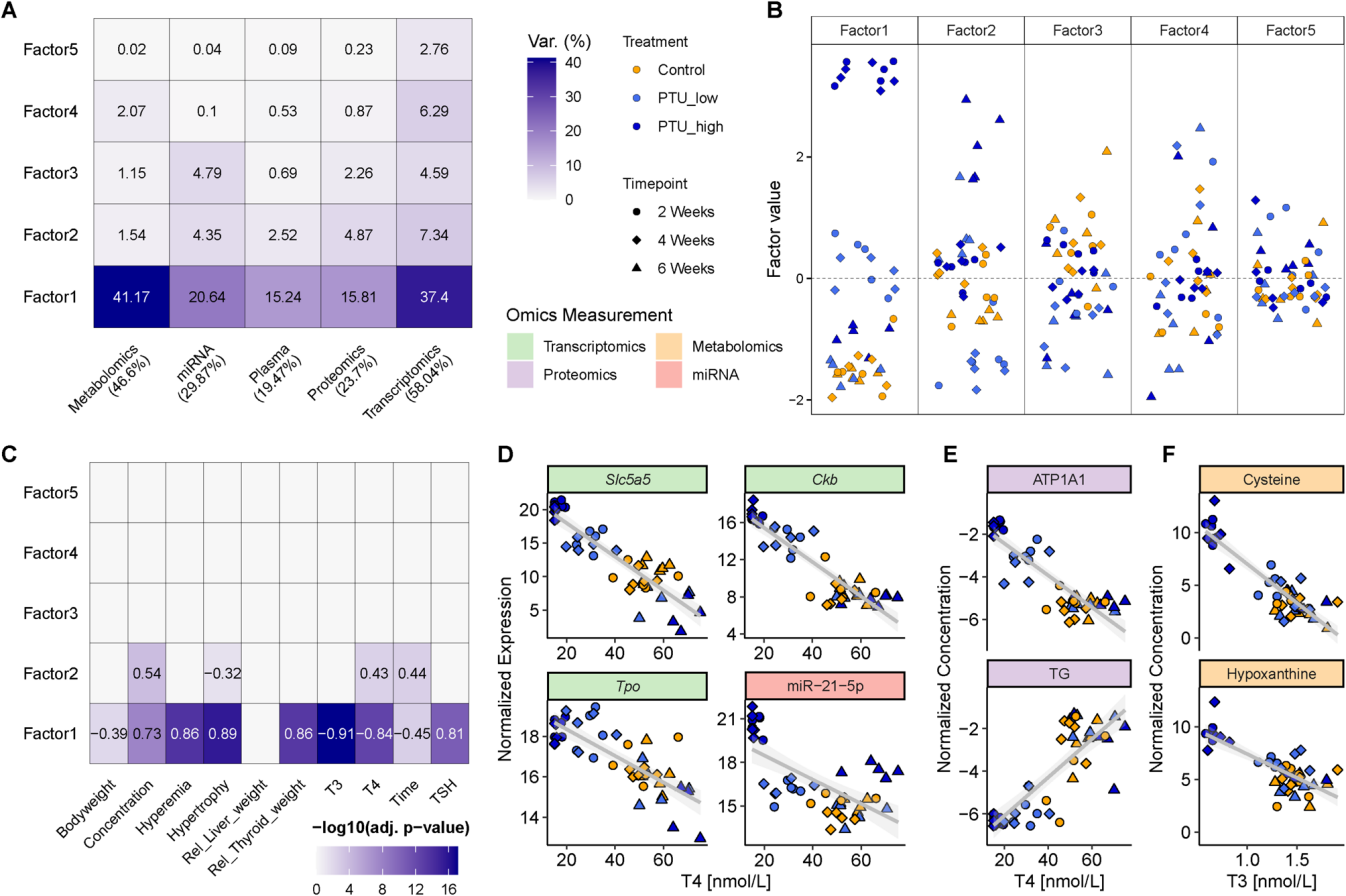
Multi-omics data integration with MEFISTO using clinical and histopathological covariates for the thyroid PTU samples. **A** Variance captured across omics layers and latent factors. The percentage of overall variance is color-coded. **B** Visualization of each factor capturing the global source of variability. **C** Heatmap showing the correlation of clinical and histopathological parameters with factor values. The Pearson correlation is written within the cell when the correlation is significant. The significance level is color-coded. **D**–**F** Correlation of normalized expression or concentration of selected (short) transcripts (D), proteins (E), and metabolites (F) with high LF1 feature weight against serum T4 and T3 levels

In Canzler et al. (2020), we concluded that simultaneous integration schemes are advantageous over sequential approaches in many aspects. We, therefore, pursued a simultaneous integration procedure utilizing the MEFISTO framework (Velten et al. 2022), which is part of the MOFA package (Argelaguet et al. 2018), for integrating the multi-omics data sets, including clinical and histopathological data as covariates. In principle, MOFA is a probabilistic factor model, and it, therefore, aims to identify principal axes of variation across multiple omics data derived from the same set of samples. We built all models with five latent factors to capture different sources of variation. The thyroid PTU model results are shown in Fig. 2. The liver Phenytoin model results are displayed in Supplemental Fig. S12.

*Thyroid PTU model.* Across all five omics layers, latent factor (LF) 1 captured the most variance in the thyroid PTU model (Fig. 2A). Each factor is a linear combination of input features that arranges samples along a one-dimensional axis centered at zero. Figure 2B displays these factor values for each sample and factor. Samples with opposite signs exhibit contrasting phenotypes along an inferred axis of variation. The strength of the effect increases with a higher absolute value. LF1 separated samples under PTU treatment from control and recovery samples in a dose-dependent manner. In contrast, LF2 separated recovery samples after high-dose PTU treatment from high-dose treated and control samples versus low-dose samples. LFs 3 to 5 captured sources of variance that did not correspond to treatment groups or clinical observations. In summary, the model identified correlated drivers across all five omics layers pointing toward a common biological source triggered by the PTU treatment.

LF1 was significantly correlated with all thyroid-relevant covariates (Fig. 2C). T3 and T4 hormone levels were, for example, anti-correlated ($r=-0.91$ and $r=-0.84$), while TSH levels were positively correlated ($r=0.81$) with *p* values $< 1e-10$. Histopathological covariates like relative thyroid weight and hypertrophy were positively correlated with LF1 ($r=0.86$ and $r=0.89$, respectively) and *p* values $<1e-13$. LF1 thus captured the cellular response to treatment with PTU across multiple molecular layers.

We investigated features with high absolute weights in each omics layer to identify the specific biological mechanisms captured by a latent factor, as these weights indicate the feature’s importance within the factor and layer. We plotted the weights of the top 20 features for each omics layer in LF1 (Supplementary Fig. S8) and LF2 (Supplementary Fig. S9). Subsequently, we selected a subset of these features that are directly linked to thyroid hormone biosynthesis, an essential process in thyroid function according to the literature. We then computed the correlation between the normalized expression or concentration values of these features and serum thyroid hormone levels, further deepening our understanding of the effects of PTU treatment on thyroid function (Fig.  2D–F). Most of these features were anti-correlated to T4/T3 hormone levels. Some examples of highly weighted features in the transcriptomics and proteomics layer include the sodium/iodide symporter (*Slc5a5* or NIS), the sodium/potassium-transporting ATPase (only the catalytic subunit ATP1A1 is shown), and the thyroid peroxidase (*Tpo*). While *Tpo* did not rank among the top features, it still showed a high weight in the transcriptomics layer. Thyroglobulin (TG), as the precursor of thyroid hormones, was positively correlated with thyroid hormone levels ($r= 0.83$) and was one of the main drivers in latent factor one in the proteomics layer. In the metabolomics layer, the amino acid cysteine and the purine derivative hypoxanthine had the highest factor weights and their concentrations were anti-correlated to thyroid hormone levels. Across LF1 and LF2, we detected several miRNAs with high feature weights that are found to play important roles in thyroid carcinoma progression, such as miR-21, miR-7, miR-15b, and miR-221.

*Liver Phenytoin model.* The first three latent factors captured substantial parts of the variance across all omics layers (Supplemental Fig. S12A). LF1 and LF3 separated high-dose from low-dose treated and control samples. Additionally, LF1 delineated high-dose recovery samples from all other samples. LF1 significantly correlated with liver infiltration, T3, and T4 hormone levels, while LF3 was significantly anti-correlated with liver hypertrophy and relative liver weight and correlated to T4 levels (Supplemental Fig. S12B). LF1 contained several features with high weights linked to an immune response in the literature. These included the HLA class II histocompatibility antigen gamma chain (*Cd74*) playing a crucial role in the presentation of antigens, the guanylate-binding gene (*Gbp2*) being a key player of host-pathogen protection, the neutrophil cytosolic factor (*Ncf1*), which is involved in inflammatory response, and *Lyz2* enabling the lysozyme activity. Furthermore, the normalized expression of these genes correlated with the number and severity of liver infiltrations (Supplemental Fig. S12D). Features with high weights in LF3 comprised transcripts and proteins known to act in the biotransformation of toxicants and, thus, in the clearance of exogenous substances. These included CYP2B1, which is part of the oxidation pathway of xenobiotics, several glutathione transferases (GSTs), UDP-glucuronosyltransferases (UGTs), and the epoxide hydrolase (EPHX1) all catalyzing conjugation reactions within the second phase of the biotransformation pathway. MiRNA features with high weights included for example miR-1224 and miR-155. The first one, miR-1224, is a known regulator of cell proliferation in hepatocytes through its anti-apoptotic target *Nfib* (Roy et al. 2017) and protects against oxidative stress-induced acute liver injury (Cheng et al. 2019). It was by far the most important miRNA feature in LF3. The latter one, miR-155, had the highest feature weight in LF1 and is known to contribute in immunity and inflammation of liver diseases by mediating the activation of immune cells leading to cytokine overexpression and immune imbalance (Xue et al. 2023). Supplementary Figures S10 and S11 display features with high weights in LF1 and LF3.

### Simultaneous multi-omics integration improved the detection of response pathways

We compared single-omics approaches with two different multi-omics enrichment approaches to assess the benefits of multi-omics data integration for identifying molecular response pathways. The results of a differential expression analysis in the transcriptomics, proteomics, and metabolomics data were used as input for multiGSEA (Canzler and Hackermüller 2020) to calculate the single-omics pathway enrichment as well as a sequential multi-omics enrichment. Therefore, Stouffer’s method (Stouffer et al. 1949) was used to combine the* p* values and generate a composite multi-omics pathway enrichment *p* value. This approach will be denoted as ’Multi-Seq’ for sequential multi-omics data integration. As a simultaneous approach, we utilized multiGSEA with MEFISTO-derived feature weights as ranking metric, denoted as ’Multi-Sim’. We computed the pathway enrichment for each latent factor separately and selected the lowest adjusted* p* value per pathway across all latent factors for subsequent comparison.

**Fig. 3:**
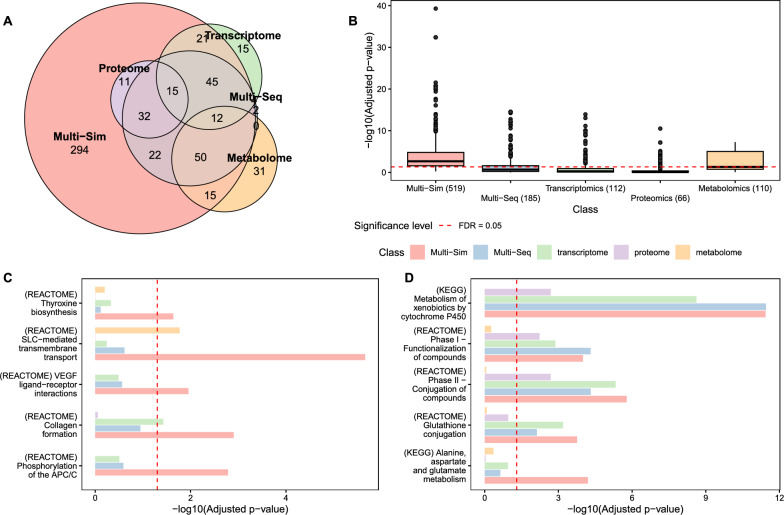
Comparison of single- and multi-omics-based pathway enrichments. All enrichments were calculated using multiGSEA. Single-omics and the log2 fold change-based multi-omics enrichment (Multi-Seq) used results of the previous differential expression analysis. At the same time, the Multi-Sim approach utilized the MEFISTO-derived factor weights as a ranking metric. Subfigures** A**–**C** indicate comparisons in the thyroid PTU model, while subfigure D is based on the liver Phenytoin model. **A** Comparison of the number of enriched pathways between single- and multi-omics pathway enrichments. **B** Boxplot indicating the significance level of all pathways that were significantly enriched with at least one enrichment approach (FDR < 0.05). **C** Comparing PTU-induced molecular target pathways in thyroid samples. **D** Comparing Phenytoin-induced molecular target pathways in liver samples

*Thyroid PTU model.* We observed a benefit of multi-omics data integration regarding the number of enriched pathways and their significance (Fig. 3A, B). In total, the Multi-Sim approach returned 522 enriched pathways (adj. *p* value $< 0.05$), the Multi-Seq approach resulted in 193 pathways, while the single-omics enrichments yielded 107, 62, and 109 pathways for transcriptomics, proteomics, and metabolomics, respectively. The Venn diagram in Fig. 3A illustrates that most pathways identified through a single-omics or the Multi-Seq approach were also enriched using the Multi-Sim procedure. Similarly, Multi-Seq also identified the majority of the single-omics-derived pathways. Multi-Sim returned an additional 301 enriched pathways that had not been found before. The p values derived through a multi-omics enrichment were generally lower compared to single-omics enrichments, and the simultaneous approach resulted in even lower p values than the sequential approach (Fig. 3B).

Subsequently, we investigated the performance of the different pathway enrichment approaches in identifying known hallmarks of PTU exposure or organ integrity in the thyroid (Fig. 3C). PTU inhibits the thyroperoxidase (Tpo) enzyme that catalyzes multiple steps in the biosynthesis of T3 and T4 (Visser 2018). Also, PTU affects the expression of the sodium/iodide symporter (SLC5A5), which mediates the uptake of iodide from blood plasma into the thyroid (Sue et al. 2012). Proper secretion of hormones from the thyroid critically depends on maintaining a specific integration of vasculature and follicular cells in angiofollicular units. The vascular endothelial growth factors (VEGFs) and their receptors (VEGFRs) are therefore critical for thyroid follicle integrity, and their expression is finely regulated by circulating TSH (Jang et al. 2017). The PTU-induced increase in TSH (see Fig. 1A) induces expression of VEGF-A and VEGFR2 and a follicular remodeling, which correlates with the observed increasing severity of follicular hyperplasia (see also Fig. 1A). In all these examples, the Multi-Sim approach detected the corresponding pathways with the highest significance. In three cases, Multi-Sim was the only method that detected a significant enrichment (Fig. 3C).

For a miRNA-based single-omics pathway enrichment, we selected the top 20 miRNAs in LF1 of the PTU-thyroid model and collected their associated target genes to perform an over-representation analysis. In total, we identified 78 significantly enriched pathways in rat and 492 pathways when using their 12 annotated human homologs (FDR $< 0.05$). In both cases, up to 15% of pathways are associated with the keyword ‘cancer’, such as ‘MicroRNAs in cancer’ or ‘Pathways in cancer’, or specific cancer types, like ‘Pancreatic cancer’ or ‘Colorectal cancer’, reflecting the previous finding that several highly important miRNAs are known oncomirs. However, there are also enriched pathways related to impaired thyroid functioning, such as TGF-$\beta$ signaling, SUMOylation, or SMAD-related pathways. For detailed results, see the corresponding Supplementary file in our git repository.^10^

*Liver Phenytoin model.* Analyzing the pathway response in the Phenytoin-treated liver samples exhibited a similar advantage of simultaneous integration regarding the number of enriched pathways and their significance. Multi-Sim identified 576 enriched pathways, while Multi-Seq and all single-omics-based enrichments found considerably less (Supplemental Fig. S16).

Phenytoin induces biotransformation enzymes in the liver. These involve members of the cytochrome P450 family for phase I and various conjugation enzymes for phase II (Sasaki et al. 2013; Piekos et al. 2018). Formation of Phenytoin-glutathione-conjugates can reduce hepatic glutathione levels, promoting liver injury (Sasaki et al. 2015). Phenytoin treatment elevates liver enzyme levels, a liver injury marker (Hussein et al. 2013). An example of these enzymes is alanine aminotransferase, pivotal in the metabolism of alanine, aspartate, and glutamate. The Multi-Sim approach detected all these except the cytochrome P450 pathways with the highest significance. For the latter, Multi-Seq performed best. The pathway on the metabolism of alanine, aspartate, and glutamate was only found to be significantly enriched by Multi-Sim (Fig. 3D).

In summary, multi-omics-based detection of known response pathways generally outperformed single-omics approaches, and simultaneous integration proved superior to sequential integration in all but one case.

Similar to the miRNA pathway enrichment in the thyroid model, we found several high-ranking cancer-related pathways in both latent factors, see Supplementary file in our git repository.^11^ Additionally, we found the FoxO signaling pathways (LF1), Toll-like receptor cascades (LF1), and various Notch signaling pathways (LF3) to be significantly enriched. FoxO proteins are critical regulators of glucose and lipid metabolism in the liver (Unterman 2018) and are also tightly linked with nuclear receptors and the formation of reactive oxygen species in response to xenobiotics (Klotz and Steinbrenner 2017). Toll-like receptors (TLRs) play a significant role in liver toxicity by mediating inflammatory responses to various stimuli (Schwabe et al. 2006). During liver injury, Notch signaling is activated in various liver cell types. It further interacts with multiple pathways to promote liver repair and regeneration (Geisler and Strazzabosco 2015).

### Multi-omics facilitates grouping

We compared the performance to cluster samples based on single- and multi-omics data. Therefore, we computed sample-wise Euclidean distances using the MEFISTO-derived factor values from single-omics and multi-omics models and calculated a hierarchical clustering. To analyze the robustness of these clusterings, we used the approximately unbiased p value support, determined through a multi-scale bootstrap re-sampling using the pvclust package (Suzuki and Shimodaira 2006).

**Fig. 4:**
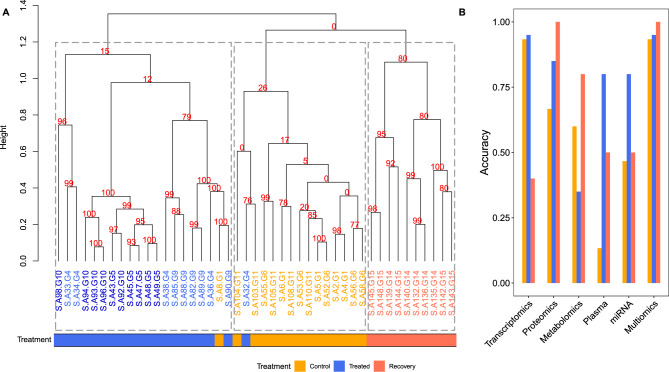
Clustering of PTU-treated samples based on the Euclidean distance of their MEFISTO-derived factor weights. **A** Cluster dendrogram of the multi-omics model. Bootstrap support for a subtree is written in red at each respective node. Samples colored in orange, blue, and coral indicate controls, PTU treatments, and recovery samples, respectively. The time and dose parameters were neglected in this clustering. **B** Comparison of cluster accuracy of single-omics and multi-omics MEFISTO models. For each model, the clustering was automatically split into the three largest subclusters (indicated by the dashed lines in **A**). Each subcluster was assigned to a particular treatment group based on the majority of its samples. The accuracy was then calculated separately for each treatment group against its respective cluster

*Thyroid PTU model.* The MEFISTO-derived multi-omics clustering resulted in three main clusters corresponding primarily to controls, treated samples, and recovery samples after treatment, respectively (Fig. 4A). An 80% bootstrap support indicated a strong recovery cluster, meaning that this specific cluster was present in 80% of all bootstrap re-samplings. Bootstrap support was lower for the cluster of treated samples. However, the subcluster covering high-dose treated samples exhibited a support of 100. The control cluster showed the lowest bootstrap support. Supplemental Figure S13 displays single-omics-based dendrograms for comparison.

The multi-omics-based clustering effectively separated the control, treated, and recovered samples. We evaluated this method against single-omics approaches by calculating how accurately samples were assigned to their respective clusters. For simplicity, we used the previously mentioned groups of controls, treated, and recovery samples, while neglecting the stratification by time and dose.

We then divided each dendrogram into three subtrees, each representing a treatment group based on the majority of samples in that group. We computed the accuracy of these assignments for each subtree describing the correct sample assignments. For instance, if a subtree designated as ‘treated’ contains 20 samples, and 18 of them are indeed treated (whether with low or high doses over 2 or 4 weeks), the accuracy would be 0.9.

The multi-omics-based clustering resulted in an overall high accuracy: recovered samples matched the recovery cluster to 100%; controls and treated samples had an accuracy of 93% and 95%, respectively (Fig. 4B). Single-omics-based clustering could not consistently recover all three groups with high accuracy: Transcriptomics failed to cluster the recovery samples correctly but exhibited comparably high accuracies for controls and treated samples. Proteomics could cluster all recoveries but showed a considerably lower accuracy for controls. The remaining single-omics models had worse clustering performance.

*Liver Phenytoin model.* Using Phenytoin-treated liver samples, we could not detect comparable results supporting the clustering abilities of MEFISTO-derived factor values (Supplemental Fig. S14). Neither the single-omics- nor the multi-omics-based approaches could reliably cluster the three groups: treated samples, recovery samples, and controls. Transcriptomics and proteomics-based clustering resulted in 57% accuracy, while multi-omics and the remaining single-omics clusterings yielded around 50%. Thus, we observed a clear drop compared to the thyroid PTU model.

### Contribution of individual omics layers

**Fig. 5:**
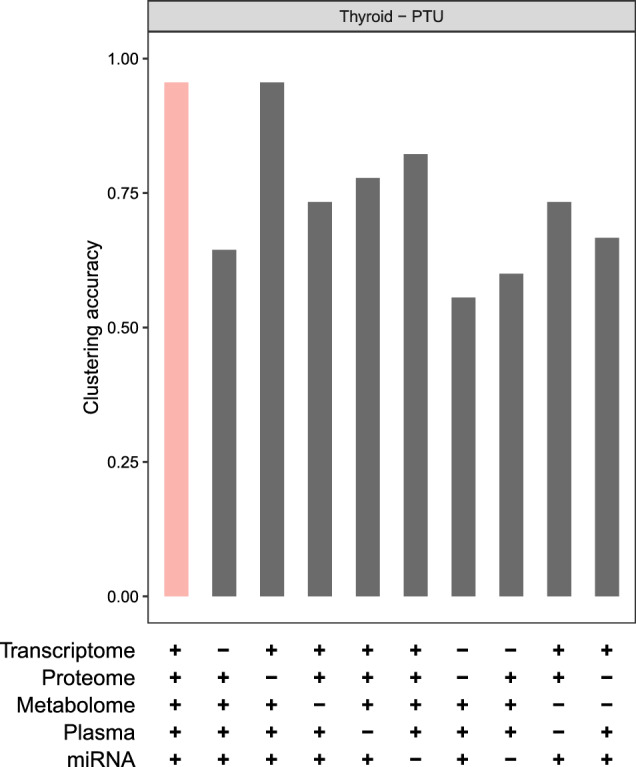
Assessing the clustering contribution of omics layers in the multi-omics thyroid PTU model. The barplot indicates the clustering accuracy of each model, as described previously. ‘+’ and ‘−’ signs below the barplot indicate which particular omics layer is present in the multi-omics model

We aimed to dissect how individual omics layers contribute to the total information of the multi-omics model. Therefore, we repeatedly performed the clustering procedure for the PTU-thyroid multi-omics model but we systematically dropped one or two single-omics layers. Starting from the complete MEFISTO thyroid PTU model, which contains five different omics layers, we built all models with four and three layers. We subsequently applied the same clustering approach as described above and compared the overall clustering accuracies of our models (Fig. 5). The complete multi-omics model resulted in the highest clustering accuracy (0.96). The transcriptomics layer provided the highest contribution of a single-omics layer: the clustering accuracy without transcriptome decreased to 0.64. Tissue, plasma metabolomics, and short RNA-seq contributed less, with clustering accuracies of 0.73, 0.78, and 0.82, respectively. Interestingly, the MEFISTO model without the proteomics layer resulted in the same clustering as the complete multi-omics model.

Removing both transcriptomics and proteomics layers from the model decreased the accuracy to 0.56. When we removed both short and long RNA-Seq data sets, the accuracy dropped to 0.60, which was lower than the four-layer models where either of both layers was missing. Removing proteomics and tissue metabolomics data resulted in a clustering accuracy of 0.67, which was also lower than for both leave-one-out models.

## Discussion

In Canzler et al. (2020), we highlighted the potential of multi-omics approaches in systems toxicology and established best practices. However, we found a lack of compliant multi-omics data sets. Here, we present a study investigating direct and indirect thyroid toxicity designed explicitly for a comprehensive multi-omics approach. We describe an experiment with paired samples across six omics layers. We provide single-omics analyses and a detailed procedure for integrating and applying multi-omics data to study the toxicological effects of two model thyroid toxins.

We discussed two alternative sampling schemes in Canzler et al. (2020): a dense sampling approach focusing on early responses and a sampling scheme that builds on repeated dosing and focuses on the steady state of a chronic signaling change. For this study, we opted for the latter to keep the design close to frequently used OECD testing guidelines, fully aware that omics layers more sensitive to immediate, transient molecular events, such as phosphoproteomics, might be less impactful at these time points.

While sex-specific differences are highly relevant in toxicology, they were beyond the scope of this study. We, therefore, included only male rats in the animal experiment, since they provide more tissue for measurements. If we had kept the overall study design but included male and female rats without increasing the number of animals in the study, we would have increased variability and would have likely compromised the clarity of our findings on the contribution of the different omics layers.

Although the administered concentrations of both compounds were selected based on previous studies, Phenytoin treatment resulted in discernible differences compared to controls only for the high dose. While this may have also been a consequence of biological variability, it also highlights the challenges and need for careful dose selection to fully capture the spectrum of toxicological responses.

### The combination of omics data with clinical readouts facilitates mechanistic insights

Based on two multi-omics models, we showed that simultaneous multi-omics data integration is beneficial compared to a single-omics analysis. The combination of clinical and histopathological readouts further facilitated the inference of mechanistic knowledge and its biological interpretation.

*Latent factors capture interpretable molecular sources of variance.* In the thyroid PTU model, latent factor one (LF1) captured the dose-dependent response of the PTU-treated samples. Essential genes, proteins, and metabolites of the thyroid hormone synthesis were associated with high feature weights and exhibited a highly significant correlation with thyroid-relevant covariates (Fig. 2). One such crucial feature is the sodium/iodide symporter (*Slc5a5*) or NIS, which received high weights in the transcriptomics and proteomics layers. This transmembrane transporter is responsible for the uptake of iodide into the thyrocytes via an active and energy-dependent transport from the bloodstream. NIS is known to be directly elevated by PTU both in terms of mRNA and protein levels resulting in an increased iodide uptake (Sue et al. 2012), see Fig. 2D (protein levels not shown). Interestingly, the expression of *Slc5a5* was downregulated during the recovery phase when T4 levels were increased compared to control samples. The following transport of iodide into the follicular colloid is mediated by PDS (gene: *Slc26a4*). However, there is neither evidence of increased expression of this transmembrane protein in the literature nor did we find high feature weights for the PDS gene or protein in LF1 of our MEFISTO model. The counterpart of NIS is the sodium/potassium-transporting ATPase, which exports sodium ions from thyrocytes into the bloodstream. Nearly all subunits of this transporter protein were associated with high feature weights in the proteomics layer, including the catalytic subunit ATP1A1, which also showed high anti-correlation of its normalized concentration with the serum T4 levels.

Other critical genes for thyroid hormone synthesis are the thyroid peroxidase (*Tpo*) and thyroglobulin (*Tg*). While *Tpo* displayed a high feature weight in the transcriptomics data set, and its expression was anti-correlated with T4 levels, TG showed a high weight in the proteomics layer, and its normalized concentrations correlated with T4 serum levels. Thyroid peroxidase is a transmembrane protein catalyzing the oxidation of I$^{-}$ to I in the follicular lumen. Thyroglobulin is the precursor of thyroid hormones produced by the iodination of its tyrosine residues.

Adrenomedullin 2 (*Adm2*) is another gene with high weights in LF1 and its expression is known to increase with rising TSH levels in a dose-dependent manner (Nagasaki et al. 2014). It acts as a potent vasodilator, expanding thyroid inter-follicular capillaries to facilitate thyroid hormone synthesis and secretion (Nagasaki et al. 2014). Some reports link serum creatine kinase expression, hypothyroidism, and TSH concentrations (mostly in human case studies like Scott et al. (2002); Ranka and Mathur (2003)), which has not been shown in rodents. We found the creatine kinase B (*Ckb*) expression upregulated in PTU-treated samples in a dose-dependent manner. Furthermore, its expression was highly anti-correlated with the T4 and T3 levels.

The purine-derivative hypoxanthine showed a high weight in LF1 and its concentration was anti-correlated to T3 and T4 levels. Thyroidal xanthine oxidase oxidizes hypoxanthine to uric acid and hydrogen peroxide, which the thyroid gland needs for iodination and thyroid hormone biosynthesis (Post and Fischer 1986).

*PTU interferes with defense mechanisms against oxidative stress.* The high feature weight of cysteine in LF1 in the tissue metabolomics layer is intriguing for several reasons. Cysteine can inhibit the oxidation activity of TPO, although this inhibition is reversible with higher iodide concentrations (Carvalho et al. 2000). In general, the cysteine metabolism is closely linked to that of glutamine (Bonifácio et al. 2021), which also has a high feature weight in LF1. These amino acids form a network capable of supplying the core metabolic pathways that underlie pivotal processes, such as reactive oxygen species (ROS), glutathione (GSH) synthesis, energy metabolism, ATP production by the mitochondrial electron transfer chain (mETC), and the generation of hydrogen sulfide (H2S), which serves as an electron donor for the mETC. Cysteine serves furthermore as a precursor to other organic compounds like homocysteine and taurine (Bonifácio et al. 2021), both with high feature weights in LF1. All four metabolites (cysteine, glutamine, homocysteine, and taurine) were significantly upregulated after 2 and 4 weeks of high-dose PTU treatment, see Supplementary Table S2(A).

GSH is an abundant non-protein thiol, serving as a crucial defense against both acute and chronic toxicities (Sreekumar et al. 2021; Li 2011). In a two-step enzymatic process, GSH is synthesized of L-glutamine, L-cysteine, and glycine. Though we could not assess GSH levels in thyroid tissue or plasma, the significant upregulation of glutamine and cysteine suggests increased GSH levels. Their antioxidant properties may explain the upregulation and high feature weights of those metabolites. The thyroid gland is vulnerable to damage from external chemicals and free radicals. Cysteine, taurine, or GSH can help shield the thyroid gland from oxidative stress and mitigate this damage (Shcherbatykh and Chernov’yants 2021; Surai et al. 2021; Kochman et al. 2021). Taurine, for example, is protective against elevated oxidative stress resulting from PTU-induced hypothyroidism (Taş et al. 2006).

Most genes and proteins involved in biosynthesis pathways utilizing cysteine, glutamine, taurine, or GSH were either unaffected or showed insignificant regulation in thyroid tissue during PTU treatment (Supplementary Table S2A). This suggests that biosynthesis may have occurred elsewhere, and these metabolites were transported to the thyroid. Intriguingly, we observed significant regulation of some of these enzymes in liver tissue, but only after 2 weeks of high-dose PTU treatment (Supplementary Table S3B). This includes the downregulation of cysteine dioxygenase (CDO) and cysteine sulfinic acid decarboxylase (CSAD), both crucial for taurine production from cysteine, and the upregulation of cystathionine $\gamma$-lyase (CTH), which is a key enzyme in cysteine metabolism. Assuming these metabolites were synthesized in the liver, we found plasma levels of these metabolites to be unaffected.

*Multi-omics integration hints at post-transcriptional regulation.* When comparing the feature weights of genes and proteins, it is apparent that some features showed high weights in both omics layers, while others revealed high weights only in one layer. The first case indicates a direct link between the regulated expression of a transcript (either upregulation or downregulation) and its measured protein concentration, following the narrative that increased or decreased transcript numbers directly result in increased or decreased protein concentrations. For the latter case, different explanations can hold true. First, due to the often smaller feature sizes of proteomics versus transcriptomics data sets, the protein corresponding to a high-weight transcript, such as Adm2 in the thyroid PTU model, may be missing in the proteomics layer. Second, upregulated transcription does not necessarily result in increased protein concentrations. Post-transcriptional regulation can heavily affects protein levels, since more than 60% of human coding genes are predicted miRNA targets (Friedman et al. 2009). Often, miRNAs (down)regulate protein levels to a modest degree (Baek et al. 2008), with exceptions up to 4-fold (Farazi et al. 2011). However, this could still be enough to alleviate the protein-level effect of measurable differential expression on the transcriptome.

The integration of short RNA-Seq data with transcriptomics and proteomics data sets might facilitate the identification of such regulatory triangles. An interesting candidate in the thyroid PTU model is miR-224-5p, which showed high feature weights in LF1 and was significantly downregulated in high-dose PTU-treated samples (log2FC and adjusted p values of features discussed here are listed in Supplementary Table S4). This miRNA targets the 3’ UTR of Type 1 iodothyronine deiodinase (*Dio1*) across mammalian species (Boguslawska et al. 2011). On the transcriptome level, *Dio1* was significantly downregulated in high-dose-treated PTU samples. A simultaneous downregulation of miR-224-5p may, therefore, prevent the complete switch-off of DIO1 protein levels. However, we did not detect DIO1 in the proteomics layer, and hence, this hypothesis remains to be verified.

A major driver in LF2 is miR-199a-5p, which is significantly upregulated in high-dose PTU samples, including the recovery phase. One of the targets of miR-199a-5p is *Snai1*. This zinc finger transcription factor is one of the best-known regulators of thyroid cancer by inducing epithelial–mesenchymal transition (EMT) through the repression of CDH1 (Wieczorek-Szukala and Lewinski 2021). We found the *Snai1* gene to be significantly upregulated in high-dose-treated PTU samples after 2 and 4 weeks but we have no data on the proteome level. CDH1 levels, however, were neither found to be differentially altered in the transcriptome nor proteome data, hinting at unaffected proteome levels of SNAI1, potentially via the role of miR-199a-5p. This would align with the recent findings that overexpression of miR-199a-5p inhibits the progression of papillary thyroid carcinoma (PTC) by downregulating SNAI1 (Ma et al. 2018).

*Basp1*, a potential thyroid tumor suppressor, inhibits cell growth and migration and induces apoptosis (Guo et al. 2016). It is targeted by miR-21-5p (human) and miR-7a-2-3p (mouse). Both rat miRNA homologs showed high feature weights in LF1 and significant differential expression, although with opposing effects. *Basp1* was significantly upregulated at the transcript level after 2 and 4 weeks of high-dose PTU treatment but downregulated at the protein level, although not significantly. This discrepancy suggests that miR-21-5p may inhibit *Basp1* translation rather than degrade its mRNA (Fabian and Sonenberg 2012; Naeli et al. 2023).

These examples indicate the importance of generating both transcriptome and proteome data to assess post-transcriptional regulation through miRNAs. However, the proteomics layer needs to provide sufficient coverage to identify links in a meaningful way.

Several miRNAs with high feature weights in the thyroid PTU model do not have a direct connection to thyroid hormone synthesis but are associated with the progression of papillary or medullary thyroid carcinomas, such as miR-21 (Zang et al. 2019; Pennelli et al. 2015), miR-375 (Galuppini et al. 2017; Romeo et al. 2018), miR-130b (Boufraqech et al. 2016), or miR-451a (Tan et al. 2022) in LF1 and miR-221 (Cai et al. 2021) or miR-199a (Ma et al. 2018) in LF2. We might assume that these miRNAs may be considered closer to the adverse outcomes than to the molecular initiating events associated with PTU exposure. Some of these miRNAs are promoters, and some are inhibitors of tumor progression. However, we did not identify coherent regulatory patterns across both types. Furthermore, we are aware that rigorous in vivo confirmation is generally missing for most (human) cancer-related miRNAs (Mockly and Seitz 2023).

Within the thyroid PTU model, we identified important features in LF1 and LF2 across different omics layers directly or indirectly involved in thyroid hormone synthesis or the progression of PTU-induced thyroid toxicity. We could show that transcriptome and proteome are mutually beneficial and that the addition of short RNA-Seq facilitates the exploration of post-transcriptional regulation. Metabolomic features illuminate the molecular response from a different angle, highlighting the benefit of combining multiple omics layers to obtain a comprehensive picture.

*Clinical and histopathological readouts facilitate inference of mechanistic knowledge.* The combined analysis of sample factor weights, the inference of functions of features with high weights, and the correlation of LFs with clinical and histopathological readouts facilitate the biological interpretation of sources of variance. For example, the discrimination of high-dose Phenytoin-treated samples (2 and 4 week treatment) from controls and recovery samples in LF3 of the liver Phenytoin model points toward a treatment-induced liver response; see Fig. S12. This is fostered by significant anti-correlations to clinical and histopathological covariates like body weight, relative liver weight, concentration of Phenytoin, and serum T4 levels. Furthermore, genes, proteins, and metabolites that are involved in the clearance of xenobiotics showed concordantly high feature weights in LF3. Liver enzyme induction in rats, especially uridine diphosphate glucuronosyltransferases (UDOGTs and UGTs), is commonly observed after exposure to exogenous compounds like Phenytoin (Bomann et al. 2021). Since T4 hormones are also metabolized by UGTs, the induction of liver enzymes by Phenytoin may contribute to the observed clearance of T4. T4 serum levels were weakly but significantly decreased after 4 weeks of high-dose Phenytoin treatment, see Fig. 1B. The systemic depletion of T4 would also result in an increase of TSH via the hypothalamic–pituitary–thyroid axis feedback loop (McClain 1989) and later on, if the concentrations are high enough and the exposure time is long enough, in thyroid follicular hypertrophy and thyroid tumors (Hill et al. 1989; Curran and DeGroot 1991). We observed a trend that mean TSH levels increased by 54% compared to controls after 4 weeks of high-dose Phenytoin exposure, although this was not significant. We furthermore found only two out of ten animals with slight follicular hypertrophy (grade 1). Those findings indicate that we captured the rat-specific thyroid hormone clearance through hepatic microsomal enzyme induction in LF3, but the administered doses were too low and/or the exposure times too short to observe more severe effects.

*Improved detection of response pathways.* The application of multi-omics pathway enrichment methods proved beneficial in terms of the number of enriched pathways and the significance of enrichment. This holds for both presented models, the thyroid PTU model and the liver Phenytoin model, where several pathways, such as the Thyroxine biosynthesis or the Alanine metabolism, were exclusively found to be significantly enriched using multi-omics approaches.

Using multi-omics methods, applying multiGSEA utilizing MEFISTO-derived feature weights (Multi-Sim) was beneficial compared to a sequential enrichment by applying multiGSEA utilizing single-omics results (Multi-Seq). This highlights the ability of simultaneous data integration to infer correlated drivers of variation across multiple omics layers.

Individual LFs identify specific sources of variation and, hence, are not directly comparable with results that originated from a global perspective, like a differential gene expression analysis. To alleviate this hurdle, we calculated the Multi-Sim enrichment for each LF separately. We selected the lowest adjusted p value to ensure that pathways belonging to different biological responses or variation (from different LFs) are optimally represented. This procedure also offers a significant advantage: The feature weights in each LF highlight the molecular response captured therein and, therefore, favor the LF-specific pathways while minimizing the noise that may be introduced when a global view is applied.

The precise statistical assessment of the enrichment methods is challenging, because an appropriate reference is missing: it is not fully known for the specific biological system under consideration which specific pathways should be affected (positive set) and which pathways should not be affected (negative set) by the treatment. As a result, the lists of significant pathways provided by enrichment methods might include pathways that are in fact not significantly impacted (false positives), or fail to include truly impacted pathways (false negatives). Although the total number of enriched pathways is elevated when multi-omics approaches are used, the benefits should outweigh the increased efforts to identify the response pathways within a more extensive set of enriched pathways. In a toxicological setting, we could argue with the precautionary principle and accept that we would rather detect a false-positive activation of a pathway than overlook it.

In addition to the multi-omics approach described above, we found that pathway enrichments based on miRNA target genes can provide additional insights. For example, in the thyroid PTU model, enriched pathways in the miRNA layer include TGF-$\beta$ signaling-, SUMOylation-, and SMAD-related pathways, which are crucial for regulating thyroid differentiation and function (Nicolussi et al. 2003; Mincione et al. 2011). The literature describes the downregulation of NIS by TGF-$\beta$ signaling through SMAD3 interference with PAX8 in a dose-dependent manner (Kawaguchi et al. 1997; Costamagna et al. 2004). Despite significantly increased expression levels of key drivers, including all TGF-$\beta$ receptors and SMAD3, we observed significantly increased NIS transcript and protein levels with unaltered PAX8 levels. However, several miRNAs targeting this signaling cascade were significantly differentially expressed and showed high feature weights in LF1, potentially modulating the transcriptome-level expression. Due to missing proteomics data for these features, this hypothesis still needs to be confirmed. For example, miR-21-5p is involved in the regulation of STUB1-mediated ubiquitination of SMAD3. More details, including involved genes, proteins, miRNAs, their interactions, and expression level changes, are given in Supplementary section S4. However, we must note that those pathway enrichments are only as accurate as the necessary annotation of miRNA target genes. Although we restricted our analysis to experimentally verified targets, there are concerns about a methodological and functional bias in such miRNA-based enrichment analysis that have to be considered when interpreting the results (Bleazard et al. 2015; Mockly and Seitz 2023).

Besides, the incorporation of miRNA target gene pathway enrichments with multi-omics tools such as multiGSEA is hindered by several technical issues. GSEA approaches utilize all genes with a specific ranking metric, whereas miRNA enrichments rely on over-representation analysis for target genes. The coverage of annotated targets may be insufficient depending on the studied organism, necessitating mapping miRNA genes to human orthologs. Additionally, frequently updated tools for miRNA pathway enrichment are often web-based, such as miRPATH-v4.0 (Tastsoglou et al. 2023), which prevent continuous integration and reproducibility.

By addressing these challenges, integrating miRNA data into multi-omics approaches can further enhance the detection and interpretation of biologically relevant pathways, potentially leading to more comprehensive insights into the molecular mechanisms underlying the studied conditions.

*Multi-omics data integration facilitates grouping.* The multi-omics thyroid PTU model clearly showed an improved ability to classify samples into the groups ‘control’, ‘treated’, and ‘recovery’. Different omics layers provided different capabilities to distinguish specific groups, and only a combination of multiple layers could discriminate most of the samples.

The results of the multi-omics liver Phenytoin model appear contradictory at first glance, since single-omics layers like transcriptomics and proteomics show better clustering abilities. However, this result is mainly caused by the overall high variance within each treatment group and the fact that low-dose treated samples showed no pronounced effects on any molecular layer. Hence, those groups cannot be reliably distinguished from controls. When the grouping procedure was re-analyzed with a reduced set of samples, including only controls, high-dose treated samples, and recoveries after high-dose treatment, we again observed a benefit of multi-omics-based clustering, which showed an elevated clustering accuracy compared to all single-omics models, see Supplementary Fig. S15.

Both models showed that measurable and quantifiable effects between treatment groups are required in each individual omics layer and that combined multi-omics-based clustering is beneficial in deriving a more stringent and reliable classification.

### Which omics layers should be used for toxicological research questions?

Finally, we evaluated the specific contributions of individual and combined omics layers on grouping. Employing a leave-one-out approach, we assessed the clustering capability of multi-omics models with one or two layers omitted in the context of PTU-thyroid samples. The complete five-layer model served as the baseline reference, and the disparity in clustering accuracy quantified the impact of individual omics layers. The transcriptomics layer had the most significant impact, followed by tissue and plasma metabolomics. Remarkably, the removal of the proteomics layer demonstrated no discernible effect. This finding is somewhat unexpected, because the single-omics PCA of thyroid samples suggested that proteomics data effectively captured PTU-associated responses (Supplementary Fig. S6C). Notably, the joint exclusion of proteomics and, for instance, transcriptomics resulted in a further decline in clustering accuracy compared to models without transcriptomics alone.

The leave-one-out results underscored that integrating multiple omics data sets enhances the model’s sample clustering capabilities. Even seemingly similar omics layers contribute distinct value to the clustering approach, as evidenced by the reduced accuracy when both metabolomics layers were removed compared to the exclusion of each metabolomics layer individually.

In summary, the specific benefits of each omics layer are not readily known beforehand and depend on the research question and the exact experimental setup. Therefore, meticulous planning of the experimental design, especially regarding applied concentrations and sampling time points, is pivotal. Late sampling time points, as applied in our experiment, infer a steady state and reliably capture transcriptomic and proteomic perturbations but may overlook rapid phosphoproteomic changes. Short RNA-Seq data, while valuable for clustering and identifying essential features, particularly in post-transcriptional regulation, need better integration with pathway databases and currently necessitate manual, in-depth analysis to unravel the mechanistic role of short RNAs.

From the presented data and analyses, we conclude that multi-omics approaches greatly facilitate the detection of molecular responses to chemical exposure at the pathway level. They are very helpful for grouping samples and thus may also have a role in supporting read-across.

### Regulatory applicability

In our research, we have demonstrated the substantial value of multi-omics data integration in toxicological studies, specifically through the lens of investigating thyroid toxicity. This approach not only deepens our mechanistic understanding but also aligns with the principles of next-generation risk assessment, which emphasizes the importance of mechanistic data to elucidate modes of action leading to adverse outcomes. Our findings advocate for incorporating multi-omics data into regulatory frameworks to enhance the predictive accuracy of risk assessments and support the identification of biomarkers for toxicological outcomes. However, the complexity of multi-omics data integration and interpretation remains a significant challenge. Further simplification and standardization of such methods are crucial to ensure broader applicability in regulatory frameworks.

The utilization of multi-layered omics data in guideline studies presents several advantages. First, it generates a comprehensive molecular profile of biological responses to chemical exposures, facilitating robust statistical analyses that can discern subtle yet significant changes across the genome, proteome, metabolome, and more. This wealth of quantitative data surpasses traditional single-layer analyses, offering a nuanced view of the molecular alterations that underpin toxicological responses. Second, the integration of omics data enables the construction of predictive models for biological pathway perturbations and modes of action. By leveraging extensive databases and bioinformatics tools, such as BASF’s MetaMapTox for plasma metabolomics, we can extract meaningful insights into a wide array of mechanistic pathways and toxicological endpoints. This approach enhances our understanding of specific toxicities and broadens our perspective to encompass a comprehensive range of biological effects.

Moreover, the unbiased nature of omics measurements, coupled with adequate biological replication, allows for an exhaustive evaluation of a compound’s bioactivity. This holistic view is paramount in identifying novel biomarkers and understanding the full spectrum of a substance’s toxicological profile. The potential for omics data to inform regulatory decisions is immense, particularly when biomarkers or panels thereof can be rigorously correlated and causally linked to adverse outcomes. Such biomarkers could facilitate future studies, offering refined tools for risk assessment and potentially reducing reliance on animal testing by providing more direct evidence of chemical toxicity.

Incorporating the evolving landscape of regulatory science, our research underscores the critical role of multi-omics integration in elucidating the mechanistic underpinnings of toxicological responses. Recognizing this potential, regulatory initiatives have begun to formalize the integration of omics data into risk assessment processes. Notably, the development of the Organization for Economic Co-operation and Development (OECD) omics reporting frameworks marks a significant stride toward harmonizing transcriptomic and metabolomic data reporting standards (Harrill et al. 2021). These frameworks aim to establish minimal reporting requirements for data generation and subsequent analysis to ensure consistency, reproducibility, and the broad applicability of omics studies in regulatory contexts. Additionally, the active development of a proteomics reporting framework signifies the OECD’s commitment to encompassing the full spectrum of omics technologies, addressing a critical gap in the current regulatory landscape.

To facilitate the implementation of these frameworks, databases such as the Gene Expression Omnibus (GEO), ArrayExpress, or PRIDE are pivotal in enforcing the adherence to minimal metadata standards. This enforcement is crucial for enhancing the quality and utility of deposited omics data, enabling more effective data sharing, re-analysis, and meta-analysis across studies. By mandating a consistent set of metadata, these databases ensure that omics data are accompanied by sufficient contextual information, thereby improving their interpretability and utility in toxicological research and regulatory assessments. Several such frameworks have been presented in recent years such as MIAME (Minimum Information About a Microarray Experiment), MINSEQE (Minimum Information about a high-throughput Nucleotide SEQuencing Experiment), MIAPE (Minimum Information About a Proteomics Experiment), or MERIT (MEtabolomics standaRds Initiative in Toxicology) (Chervitz et al. 2011; Viant et al. 2019).

If coupled with data submission requirements, the establishment of OECD’s omics reporting frameworks would represent a significant transformative shift toward a more transparent, standardized, and impactful use of omics data in the regulatory arena. However, we are aware that data from regulatory applications for real chemicals are frequently unpublished. This shift would not only enhance the robustness and reliability of toxicological assessments but would also pave the way for the identification of novel biomarkers and the development of new risk assessment models. As these frameworks evolve and expand to include additional omics layers, such as proteomics, they will further empower regulatory bodies to leverage the vast potential of multi-omics data, driving forward the principles of next-generation risk assessment. This holistic approach to data integration and standardization is poised to significantly advance our understanding of chemical toxicities and to inform safer chemical design, regulatory decisions, and, ultimately, the protection of public health and the environment.

## Supplementary Information

Below is the link to the electronic supplementary material.

Supplementary file 1 (pdf 2529 KB)

## Data Availability

Demultiplexed fastq files of short and long RNA-Seq experiments were deposited at NCBI SRA with BioProject identifier PRJNA695243. Proteomics data sets were uploaded to PRIDE with project ID PXD026835. Phosphoproteomics data sets were also uploaded to PRIDE with the ID PXD030254. Tissue metabolomics data sets were deposited at Metabolomics Workbench with StudyID ST002023. The plasma metabolomics data set was submitted to Zenodo with the DOI 10.5281/zenodo.5900664.

## Footnotes

1 https://www.ncbi.nlm.nih.gov/bioproject/PRJNA695243/.

2 https://www.ebi.ac.uk/pride/archive/projects/PXD053208.

3 10.5281/zenodo.5900664.

4 10.21228/M8MD8N.

5 https://www.ebi.ac.uk/pride/archive/projects/PXD030254.

6 https://codebase.helmholtz.cloud/department-computational-biology/xometox/process_integrate_omics_data

7 https://www.bioinformatics.babraham.ac.uk/projects/fastqc/

8 10.5281/zenodo.12626180

9 https://github.com/HKeyHKey/Canzler_et_al_2023

10 https://codebase.helmholtz.cloud/department-computational-biology/xometox/process_integrate_omics_data/-/blob/master/miRNA_pathway_enrichment/01_thyroid_miRNA_pathway_enrichment.Rmd

11 https://codebase.helmholtz.cloud/department-computational-biology/xometox/process_integrate_omics_data/-/blob/master/miRNA_pathway_enrichment/02_liver_miRNA_pathway_enrichment.Rmd
