# Supplementary material for: Evaluating the performance of multi-omics integration: a thyroid toxicity case study
Source: Arch Toxicol. 2024 Oct 23;99(1):309–32. doi: 10.1007/s00204-024-03876-2 (PMC11742338; doi:10.1007/s00204-024-03876-2)
Supplement: Supplementary file 1 — (pdf 2529 KB) [file 204_2024_3876_MOESM1_ESM.pdf]

Noname manuscript No.
(will be inserted by the editor)

Supplementary Material

Evaluation of the performance of multi-omics integration in the study of direct and indirect thyroid toxicity.

Sebastian Canzler · Kristin Schubert · Ulrike E. Rolle-Kampczyk · Zhipeng Wang · Stephan Schreiber · Hervé Seitz · Sophie Mockly · Hennicke Kamp · Volker Haake · Maike Huisinga · Martin von Bergen · Roland Buesen · Jörg Hackermüller

Received: date / Accepted: date

S. Canzler · K. Schubert · Z. Wang · S. Schreiber · U. E. Rolle-Kampczyk · M. von Bergen · J. Hackermüller
Helmholtz Centre for Environmental Research - UFZ, 04318 Leipzig, Germany
E-mail: joerg.hackermueller@ufz.de

H. Kamp · V. Haake
BASF Metabolome Solutions GmbH, 10589 Berlin, Germany

M. Huisinga · R. Buesen
Experimental Toxicology and Ecology, BASF SE, 67056 Ludwigshafen, Germany

H. Seitz · S.Mockly
Institut de Génétique Humaine UMR 9002 CNRS-Université de Montpellier, 34396 Montpellier Cedex 5, France

S1 Animal experiment

S1.1 Experimental design

Two well-described test chemicals, i.e., Phenytoin and Propylthiouracil (PTU), were administered via the diet at each two concentrations to male Wistar rats for 2 weeks and 4 weeks. In addition, a recovery period of 2 weeks without test substance application was included after the 4 weeks treatment in order to monitor reversibility of the induced effects. All test groups including the control groups consisted of each 10 male Wistar rats. Phenytoin was administered at concentrations of 300 and 2400 ppm, PTU was administered at concentrations of 5 and 50 ppm. Control animals received maintenance diet only without inclusion of any test substance. A graphical summary can be seen in Figure S1. The study was performed to generate tissue-based samples for subsequent omics data generation. The animal facility that all animal work was performed in holds a certificate from the International Association for Assessment and Accreditation of Laboratory Animal Care (AAALAC). Furthemore, the animal studies were performed with the approval of the local regulatory agencies (Nr: 23 177-07/G 19-3-061).

Food consumption and body weights were determined weekly. All animals were checked daily for any abnormal clinically signs. Abnormalities and changes were documented for each animal. Detailed clinical examinations in an open field were conducted prior to the start of the administration period and weekly thereafter. During the course of the study, blood samples were taken for metabolome analyses and examination of thyroid hormone levels (T3, T4 and TSH; all treatment groups). At necropsy, liver and thyroid glands' weights were determined, and histopathological examinations of these organs were performed. In addition, tissue samples were isolated for performance and assessment of transcriptomics, proteomics, and metabolomics.

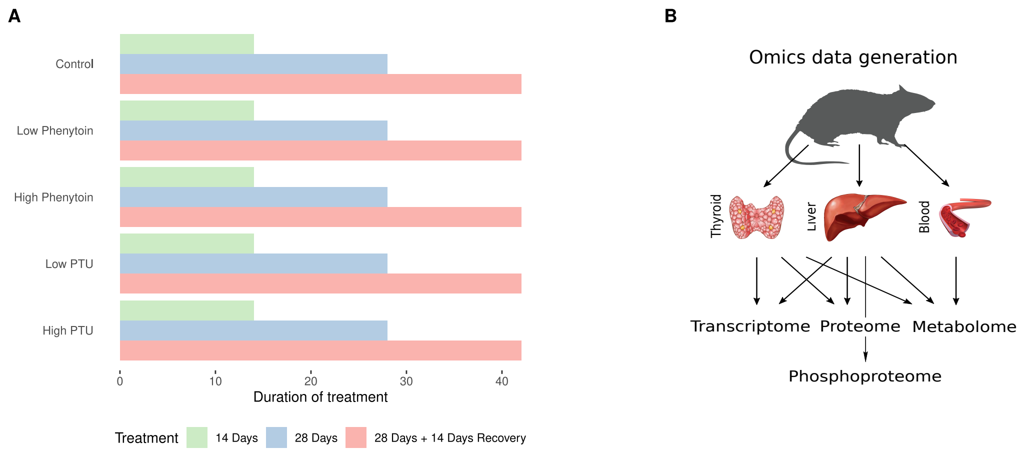

Fig. S1: Graphical summary of the animal experiment. (A) Experimental design of the study. In total, we have 15 different treatment groups based on two compounds with a low and high dose and three different sampling time points: two and four weeks of treatment and an additional two weeks of recovery. Phenytoin was administered at 300ppm (low) and 2400ppm (high) and PTU was administered with 5ppm (low) and 50ppm (high). (B) Long and short RNA-Seq, proteomics, and tissue metabolomics data sets were generated from thyroid and liver samples for each of the treatment groups. Phosphoproteomics data sets were exclusively generated from liver samples. Additionally, plasma metabolomics data sets were generated from all organisms.

S1.2 Clinical observations

Food consumption was lower in groups treated with high-dose PTU for four weeks and four weeks with an additional two weeks recovery starting from the second week of treatment onwards but

turned back to values comparable to the control during the recovery phase. Body weight loss was observed during the second half of treatment in those same treatment groups. The animals of the recovery group started to gain weight after test substance administration stopped. After four weeks of treatment, one animal showed piloerection during study days 21-23. Food consumption and body weight data were not significantly influenced by low-dose PTU treatment.

Food consumption was reduced during the first one or two weeks of treatment in groups treated with high-dose Phenytoin. Body weight loss was observed during treatment for four weeks and four weeks treatment with additional recovery. The animals of the recovery group started to gain weight after test substance administration stopped. No treatment-related changes in food consumption or body weight development were noted for the low-dose Phenytoin treatment group.

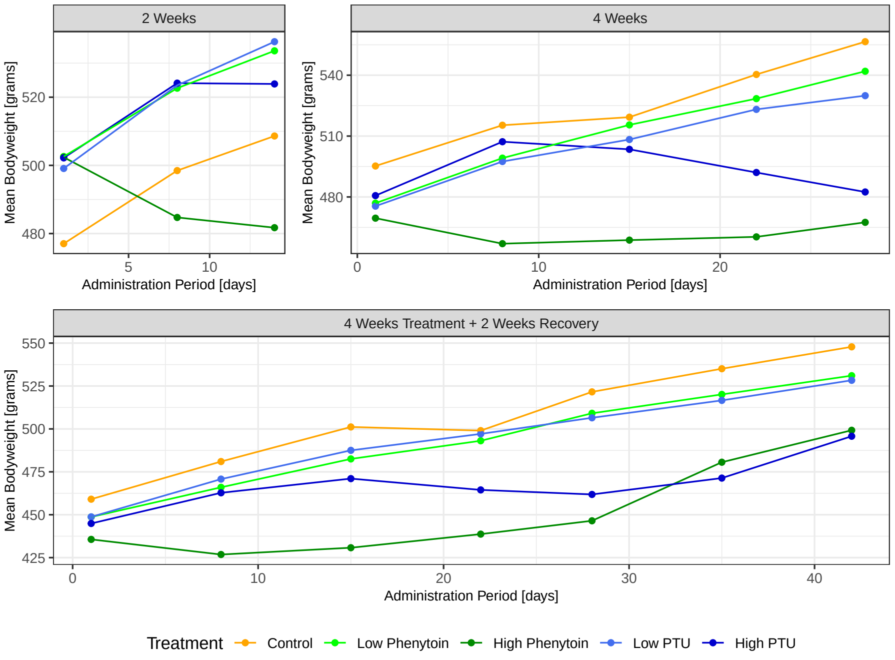

Fig. S2: Mean body weights of rat during the animal experiment. The measurements have been splitted into three groups of 2 weeks treatment, 4 weeks treatment, and 4 weeks with a subsequent 2 weeks of recovery. The body weights are averaged over all 10 animals per treatment group.

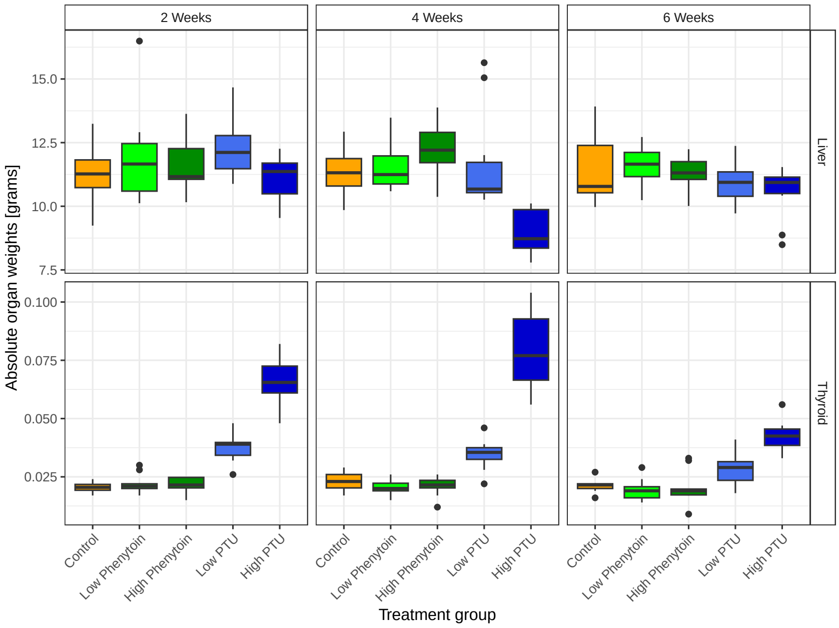

Fig. S3: Absolute organ weights of rat liver and thyroid. The measurements have been splitted into groups of 2 weeks treatment, 4 weeks treatment, and 4 weeks with a subsequent 2 weeks of recovery.

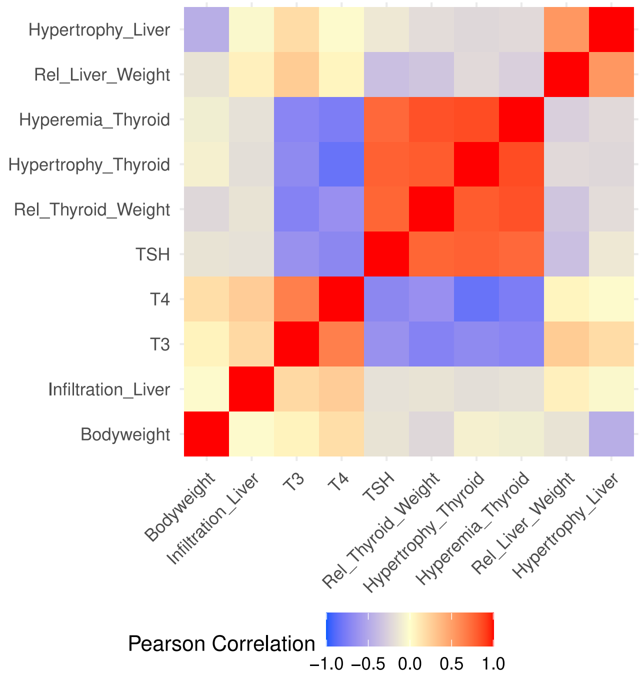

Fig. S4: Correlation heatmap of clinical and histopathological readouts of all 150 rats. Those readouts where used to calculate pairwise Pearson correlation. Euclidean distances of correlation values have been used to cluster the clinical and histopathological covariates.

S2 Omics data generation

Group	Treatment_Conc.	Duration	Liver RNA QC RIN value Cage-A					Liver RNA QC RIN value Cage-B				
			A..1	A..2	A..3	A..4	A..5	A..6	A..7	A..8	A..9	A..10
1	Vehicle_0	14	6.1	8.1	5.9	7.6	6.8	6.9	6.2	7.2	4.8	5.4
2	Phenytoin_300	14	4.8	7.7	7	7.3	7.8	7.6	7.4	7.5	5.3	7.3
3	Phenytoin_2400	14	6.2	8.4	7.9	5.7	6.9	5.1	5.3	7.4	6.4	7.8
4	PTU_25	14	6.6	8.3	8.4	7.9	7.8	7.9	5.8	8.3	7.5	6.7
5	PTU_500	14	4.1	7.3	7.3	7.2	8	6.1	7.7	7.8	7.4	6.5
6	Vehicle_0	28	3.1	7.1	7.5	5.5	7.5	7.2	5.3	7.1	5.7	5.5
7	Phenytoin_300	28	5.7	7.2	6.8	6.8	7.3	7.8	7.6	7	6.5	7.1
8	Phenytoin_2400	28	5.4	7.8	8.5	7.6	5.9	8.2	7.4	7.3	6	7.6
9	PTU_25	28	5.7	7.3	7	7.1	7.2	6.9	5.1	7.6	7.8	7.2
10	PTU_500	28	3.6	7.7	7.9	7.4	6.4	7.8	6.3	8.5	7.3	6.7
11	Vehicle_0	42	5.5	6.8	7.7	7.4	7.3	4.9	6	7	6.8	6.8
12	Phenytoin_300	42	4.1	7.3	5.8	7.3	5.6	7	7.1	6.9	6.4	6.9
13	Phenytoin_2400	42	4.7	8.1	7.4	6.8	5.1	5.2	7.3	6.7	5.7	6.5
14	PTU_25	42	4.8	5.6	4.6	4.2	6.4	6.3	3.7	5.5	6.5	7.4
15	PTU_500	42	6.8	8.0	7.2	7.5	7.5	5.6	6.1	6.6	6.4	5.4

Fig. S5: RNA integrity numbers (RIN) of all 150 liver samples. RIN values range from 1 to 10, with 10 being the highest. We aimed at selecting samples with RIN value greater than 6 to ensure sufficient quality. Five out of ten animals have been selected from each treatment group for subsequent omics data generation and are marked with black rectangles.

Table S1: Correspondence table for sample-specific short RNA-Seq barcodes. Sample ID denotes the animal number in the animal experiment. 3´ adapters used in the short RNA-Seq experiment had the pattern `NNNNTGGAATTCTCGGGTGCCAAGGAACTCCAGTCACXXXXXXXXXATCTCGTATGCCGTCTTCTGCTTG`.
`N` indicate randomized adapter ends, while the X-pattern indicate the sample-specific barcodes listed here.

Sample ID	Thyroid tissue Index 1	Thyroid tissue Index 2	Liver tissue Index 1	Liver tissue Index 2	Sample ID	Thyroid tissue Index 1	Thyroid tissue Index 2	Liver tissue Index 1	Liver tissue Index 2
2	CGCGAGAC	TTGTCGAC	CAGAGCTC	GCAATTGC	76	CTGCGGTA	GATTGGAG	CCACAGCA	GAGTCAGG
4	GAGATCGG	AAGGAGCG	TGCGATAG	CACACATG	80	GTCTTGCT	GTCTATCG	AAGGCAAT	AATTGAGA
5	CGCACTTA	CTCGAAGC	CCATTGAC	CAGATTCT	82	CTGACAGT	AATACAGG	TGCCGTTA	TGAACTAG
6	GATGTCAG	GTTAGAAC	AAGTGCTA	AATTATGC	85	GAATTGAA	TACTCTGT	TTGTGATG	CAAGTATT
8	CTACTTCG	CGAACTGT	GTACATAG	TTCATACG	88	CGTCGTCA	TGGTGGTA	TTGTATTA	AATACTTC
12	GATTACTC	CATGTCTC	TGGCAAGT	GACATTGT	89	AAGTAAGT	AATAGATT	CACTTGGC	ACGAGTCT
15	ACTCAGAC	CGACTATA	TGTGCCTT	GCGGCCAG	90	TGACGGAA	TTCTCTCA	TTGACAAG	AGATAATC
16	AAGAGTTG	AATATTGA	CCAAGGTA	CCTCGTAT	92	GAATACGG	GCACACAA	CAAGCTTG	CTATGACT
17	TTATACAA	TTCTCAAT	TGTGAACG	CGAAGATT	93	CTTATCAG	TGTGAGTC	TTGAGCAC	GTCCTAAC
18	GATTAGGA	GTGGAACG	CAACGTAA	TCCTGTCT	94	TCCGCGTC	GTCGCAGG	CGTATTCG	CCATATGC
22	CTCTGGCG	AGTTACGG	TGTTAATT	AGGACAAC	96	CTGAGTGC	GCATCACA	TACGCATA	CCAGCCTA
23	GATACCTA	GCGAGATC	CCTTAGTA	AGCCTCTG	98	TCCTAACG	TCCAACTA	CTTAACTC	GCCATAAG
25	CGCGTATC	AGCGTACG	TGACTCAG	GCACTCGG	103	CGAGATAA	TGGTCACA	AAGGCCTG	AATTGTCT
28	GATACGAT	CTTGGTAC	CAGTGCCG	CAACACTG	104	TCGCGATT	CCAAGCAC	TGCAGGCA	TTCCAATC
30	CTGGAGCT	CGCAGCTG	TGAACGTC	GCGAGTGA	105	CTAGTTAT	CCTTGATG	TACGGTCG	CATCTTGC
32	GAACGATA	ACGGCACA	CAGGTTAA	CCAGCAAG	108	TCGCACTC	GATGGTAA	CGGTTCGC	CGCTCAAC
33	CGGTCCAT	GCGGTGTG	AAGGATTC	AATTACCA	110	CTATTATC	CCTCCGAG	CTAACCAA	GTGTTGTA
34	GAACTGGC	CGATACTA	CACATGCA	GCCGCCGA	112	AAGTACAG	AATAGCAA	TATGTGTG	GAATTATG
36	CGGTGAGA	CAGCTACA	TGGTCCGG	CCACCGGC	114	TGAATGGA	TTCGCTTG	CGTTAACA	AGATGAAG
38	AAGACATA	AATATGCT	CACCTCGA	CTCTAATA	116	TCGAAGCT	GCCACGCG	TAACTTGG	GCATGGCT
43	TTATGTAT	TTCTCCGC	TGGTATCT	GCTCCTCA	117	CTCATAAT	GCGCTGAC	CGGCGCAC	GTGTACGT
45	GAACAGAT	CTATATTG	CACGATAT	CGCGTTGT	118	TCTTAGGC	TACACAAC	TAAGCGTT	CATACGGC
47	CTTGGCCT	CAGGAAGG	TGGATCTA	AAGGTTGG	122	CTCTACAC	GTATGGTC	CTTAAGCA	CGTTCCTG
48	GAACATTC	GCCGTGCA	CAAGACCA	GTAGTGTT	123	TCTAGGTT	AGTCACAA	AAGCATAT	AATTGGTC
49	CTGATATA	GAGTTGCG	TGTCTTGT	CTTCCTTG	124	CGTTCTGC	CCGCTAAT	TTCTTAGA	TTACAACT
52	GAAGCGAG	AACCTCAC	CACATTAC	CACGCCGG	127	TCACTACG	CCTAGTGG	TAAGGCGG	AGACTAGG
53	CGGCCTCT	AACCATGG	AAGTCCGC	AATTAGAT	128	CTTCACGA	GCTAGAGT	CTTCTGTC	GCTACTAC
55	GAAGTCGA	TCACTGTT	TTCAGGAG	TTAAGCTA	132	TCAGTGAG	CGTGCAGG	TAATCACG	GACTTCTT
56	CGGCCATG	GACTGAGC	TTCCTCAT	GTCCTGTG	135	CTTCTTAA	CGTAGTTC	TTGCTGGC	CAGACCGA
58	GAAGTTAC	GTCCAAGT	TTCAACGG	CTGTTAGG	136	AAGTTATC	AATTAATG	ACCGACAG	CGTATCGA
62	CTTAGAGA	CCGATGCG	CAAGGAAC	ACGGAGAT	139	TGACCGCG	TTAATTCA	TGTCTATC	CCTACCGT
65	AAGACTGT	AATATCTG	TTGCCGTT	ACGACCGG	140	TCAGACTT	TGTGCAAC	ACCTATAT	CTAATCTT
66	TTCGGAAC	TTAGCTAA	CACGCACT	CGAATAGC	142	TCATGCGT	CCGACGAC	TGTCGTAG	ACGATGGC
67	GAAGATCG	GCTTCTTG	TTGCGTTC	TGGCTGTG	143	CTTGCGGA	CTCCGCGC	ACCTAAGC	CTAGGCAG
70	CGGAGGCT	CGTTATAT	CACTAGTT	ACTCTATG	144	TCAATCCT	GCCACCTC	TGGTTAAG	TTGGAGGA
72	GAAGAAGC	TGCGCGAT	TTGCGACA	CGGAAGGA	145	CCGGCTGA	CCTCTGGA	AAGCACGA	AATTCTAA
73	CTGCGAAG	CGACATGG	CAACGGTC	ACTCGGCG	148	TGCCGCCG	GACACAGA	TGAGTTCG	TTAAGAGT
74	GAATCTGA	GAAGCCTC	TTGGACAG	GCTCTAGC					

S2.1 Plasma metabolomics

MS-based metabolite profiling of blood plasma was performed according to the following protocol: Targeted metabolites were extracted from 60 μl rat plasma by adding 1500 μl extraction buffer (methanol, dichloromethane, water and toluene (93:47:16,5:1, v/v) buffered with ammonium acetate) using a ball mill (Bead Ruptor Biolab). Internal standards were added to the extraction mixture. After centrifugation, (12000 rpm, 10min, 12°C) a 100 μl aliquot of the extract was subjected to LC-MS/MS. Thus, 2.5 μl of the extract were injected each for reversed-phase and hydrophilic interaction liquid chromatography (ZIC - HILIC, 2.1 x 10mm, 3.5 μm, Supelco) followed by MS/MS detection (AB Sciex QTrap 6500+) using the positive and negative ionization mode. For reverse-phase high performance liquid chromatography (RP-HPLC, Ascentis Express C18, 5cm x 2.1mm, 2,7μm Supelco), gradient elution was performed with water/methanol/0.1 M ammonium formate (1:1:0.02 w/w) and methyl-tert-butylether/2-propanol/methanol/0.1M ammonium formate (2:1:0.5:0.035 w/w) with 0.5% (w%) formic acid (0 min 100% A, 0.5 min 75% A, 5.9 min 10% A, 600 μl/min). HILIC gradient elution was performed with (C) acetonitrile/water (99:1, v/v) and 0.2 (v%) acetic acid and (D) 0.007 M ammonium acetate with 0.2 (v%) acetic acid (0 min 100% C, 5 min 10% C, 600 μl/min). A second aliquot of the extract was mixed with water (3,75:1, v/v) resulting in a phase separation. The polar (upper phase, 400 μl) and lipid (lower phase, 90 μl) were used for GC analysis. Both phases were analyzed with gas chromatography-mass spectrometry (GC7890-5975 MSD, Agilent Technologies) after derivatization as described in Roessner et al. (2000). Briefly, the non-polar fraction was treated with methanol under acidic conditions to produce fatty acid methyl esters that were derived from both free fatty acids and hydrolysed complex lipids. The polar and non-polar fractions were further derivatized with O-methyl-hydroxylamine hydrochloride to convert oxo -groups to O-methyloximes, and subsequently with a silylating agent (N-methyl-N-(trimethylsilyl) trifluoroacetamide). For GC analysis, 0.5 μl of the derivatized phase-separated extract were used each for analysis of the polar and lipid fractions. Steroids hormones, catecholamines and their metabolites were measured by online SPE-LC–MS/MS (Solid phase extraction -LC–MS/MS) (Yamada et al., 2002; Zhang et al., 2011). All samples were analyzed once in a randomized analytical sequence design to avoid artificial results with respect to analytical shifts. For GC-MS and LC-MS/MS profiling, data were normalized to the median of reference samples (ultrapools) to account for inter- and intra-instrumental variation. The ultrapools were generated from a large collection of rat plasma that was then aliquoted and freeze-dried for later use. For sample analysis aliquots of this material were redissolved and extracted in parallel to the study samples. In plasma, 495 semiquantitative metabolites could be analyzed using the single peak signal of the respective metabolite and a normalization strategy according to the patent WO2007012643A1 (Walk et al., 2011). Of those 495 analyzed metabolites, 454 are chemically identified, and 41 are structurally unknown.

S2.2 Tissue metabolomics

Metabolite measurements were processed in the QTRAP 5500 system (AB SCIEX) equipped with the LC 1290 Infinity system (Agilent). As for the FIA part, neither oven temperature nor columns were used, and only solvent B (290mL methanol +1 ampule FIA Mobile Phase Additive) was used for the injection. The flow rate was stabled at 0.03 ml/min for 1.6 min, and increased to 0.2 ml/min in 0.8 min and maintained for 0.2 min, then down to 0.03 ml/min in 0.2 min. The compound-specific MRM transitions were determined in positive ionization mode.

For the LC part, a MxP Quant 500 Column System (Biocrates Life Science) was equipped for use and heated by setting the oven temperature to 50°C. The substance analysis via specific MRM transitions, and LC split into a part measured in positive (LC1) and a part measured in negative ionization mode (LC2). Solvent A (2000 mL water + 4 mL formic acid) and B (2000 mL acetonitrile + 4 mL formic acid) were used as eluents.

Gradient LC1: 0-0.25 min (0.8 ml/min, 0% B) , 0.25-1.5 min (0.8 ml/min; 0-12% B), 1.5-2.7 min (0.8 ml/min; 12-17.5% B), 2.7-4 min (0.8 ml/min; 17.5-50% B), 4-4.5 min (0.8 ml/min; 50-100%

B), 4.5-4.7 min (0.8-1 ml/min; 100% B), 4.7-5 min (1 ml/min; 100% B), 5-5.1 min (1 ml/min; 100-0% B), 5.1-5.8 min (1-0.8 ml/min; 0% B).

Gradient LC2: 0-0.25 min (0.8 ml/min, 0% B), 0.25-0.5 min (0.8 ml/min; 0-25% B), 0.5-2 min (0.8 ml/min; 25-50% B), 2-3 min (0.8 ml/min; 50-75% B), 3-3.5 min (0.8 ml/min; 75-100% B), 3.5-4.7 min (0.8-1 ml/min; 100% B), 4.7-5 min (1 ml/min; 100% B), 5-5.1 min (1 ml/min; 100-0% B), 5.1-5.8 min (1-0.8 ml/min; 0% B).

S2.3 Phosphoproteomics

Phosphoproteomics was conducted on liver samples only. The previously described SP3-based proteomics approach was applied to get reduced, alkylated and TMT labeled peptides before phosphopeptide enrichment with the obtained TMT mixes. To facilitate the enrichment of a suitable number of phosphopeptides, we increased the starting protein input to 200μg for each sample and adjusted the reagents for the subsequent sample preparation accordingly. The phosphopeptides enrichment was accomplished using two sequentially combined phosphopeptide enrichment kits, i.e., High-Select TiO2 Phosphopeptide Enrichment Kit (Thermo Fisher, USA) and High-Select Fe-NTA Phosphopeptide Enrichment Kit (Thermo Fisher, USA) as described in (Großkopf et al., 2021). Thereby, the phosphopeptides were enriched according to the manufacturer's instructions with some exceptions. Flowthroughs of this workflow were saved and used as complementary proteome, which was analyzed like the proteome.

For the LC-MS/MS measurement of the enriched phosphopeptides, the same instrument setup as for the proteome was used, and only LC gradients and some parameters were adjusted to facilitate identification of phosphopeptides. The combined TMT-labeled samples were loaded on a trapping column for 3 min (Flow rate: 5 μl/min; mobile phase: 98% water/2% ACN/0.05% trifuoroacetic acid). Subsequently, they were separated on an analytical column (Flow rate: 300 nl/min; mobile phase A: 0.1% formic acid in water, mobile phase B: 80% ACN/0.08% formic acid in water). A 180min gradient was used stepwise adjusting the percentage of mobile phase B: 2 min with 4% B, 77.5 min from 4% to 18% B, 37.5 min from 18% to 30% B, 30 min from 30% to 55% B, 5 min from 55% to 99%, 10 min with 99% B, 5 min from 99% to 4% B, 2.5 min with 4% B, and 7.5 min loading column wash in the end. Spectra were acquired according to the same parameter setup as in the proteome part with the following exceptions: for full MS spectra, maximum injection time (IT) to 150 ms. The top 15 precursor ions were isolated and fragmented, maximum IT to 150 ms.

S3 Omics data analysis

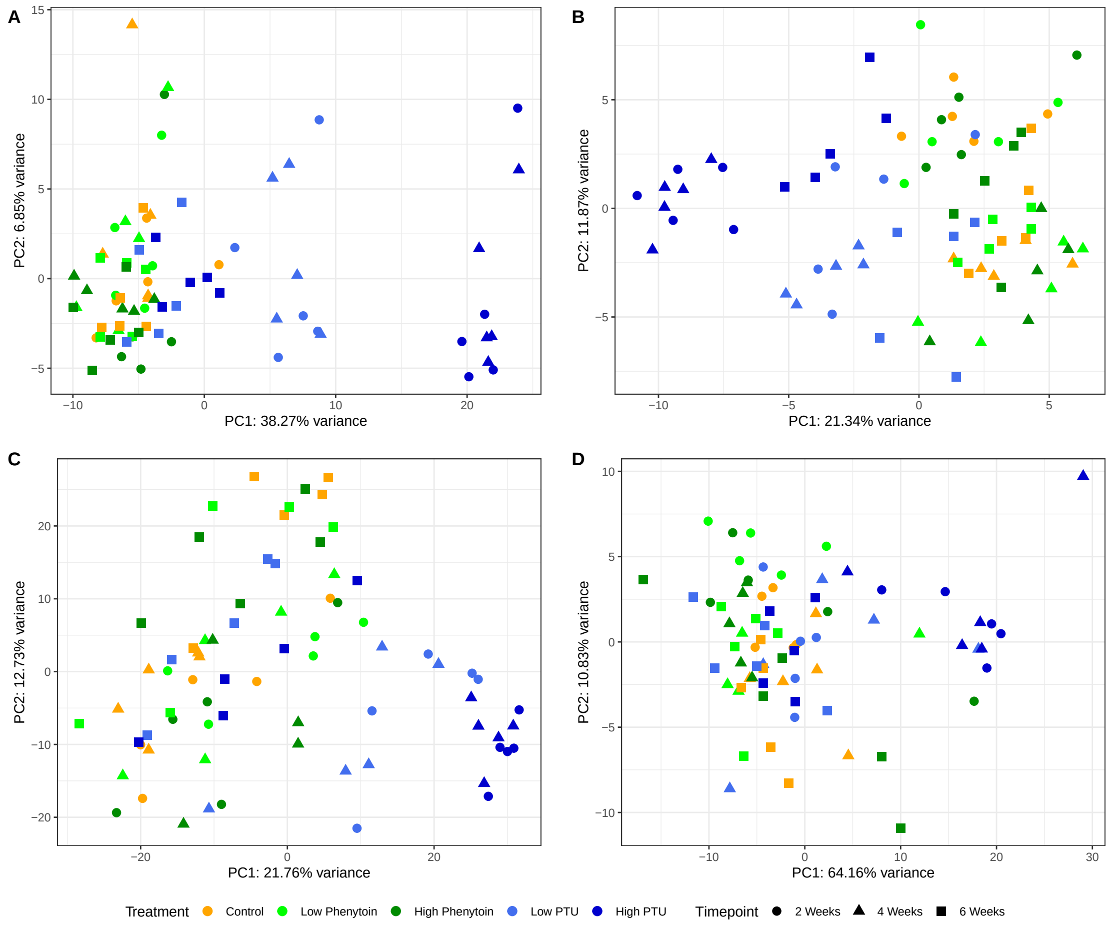

Fig. S6: Principal component analysis of thyroid samples. (A) PCA of the transcriptomics data set. (B) PCA of the short RNA-Seq data set. (C) PCA of the proteomics data set. (D) PCA of the tissue metabolomics data set. In each plot, colors represent different treatment groups based on their treatment. Controls are colored in orange, Phenytoin treated samples are green and PTU treated samples are colored in blue. Different shapes represent the duration of the treatment.

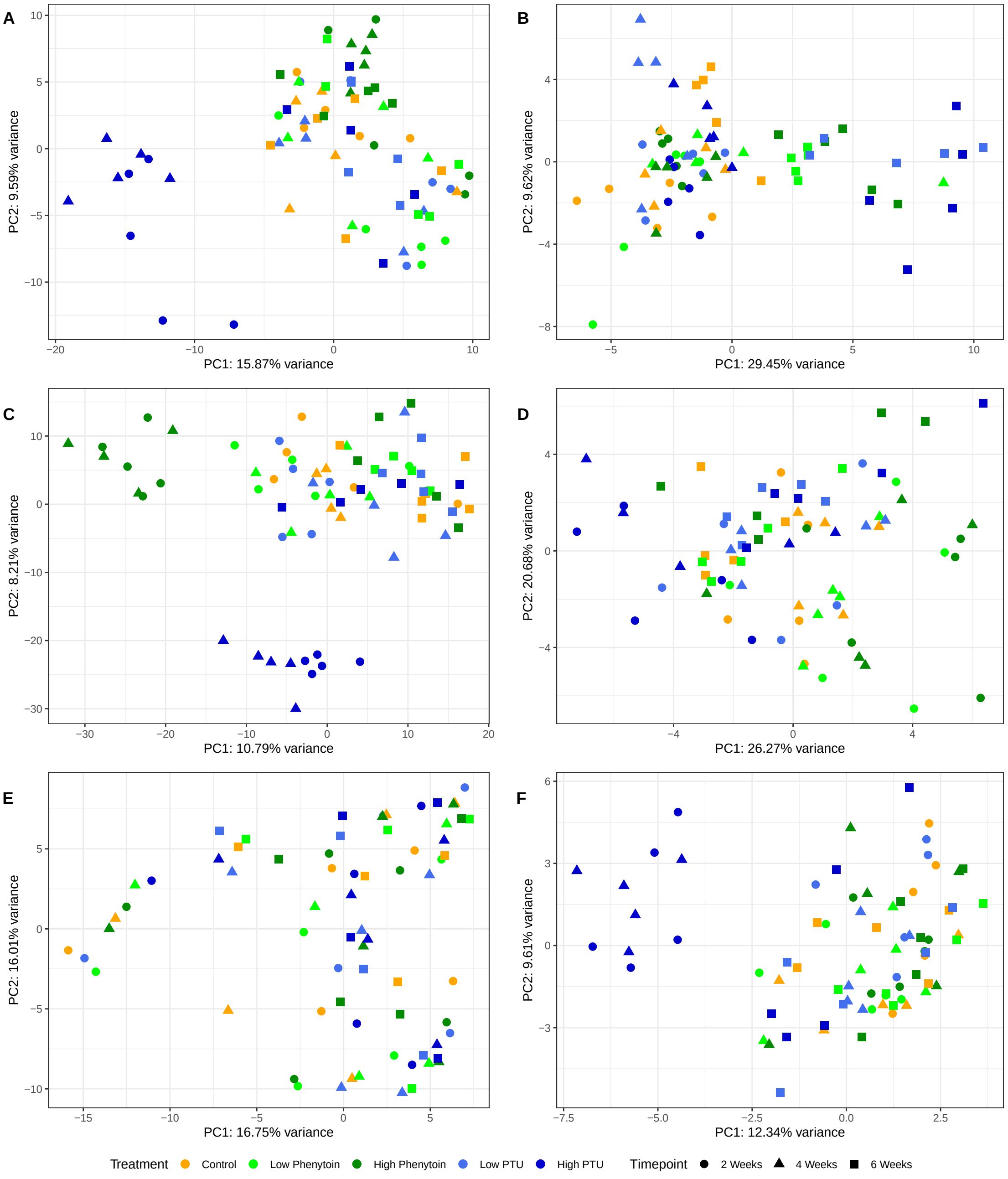

Fig. S7: Principal component analysis of liver samples. (A) PCA of the transcriptomics data set. (B) PCA of the short RNA-Seq data set. (C) PCA of the proteomics data set. (D) PCA of the tissue metabolomics data set. (E) PCA of phosphoproteomics data. (F) PCA of plasma metabolomics data. In each plot, colors represent different treatment groups: Controls are colored in orange, Phenytoin treated samples are green and PTU treated samples are colored in blue. Different shapes represent the duration of the treatment.

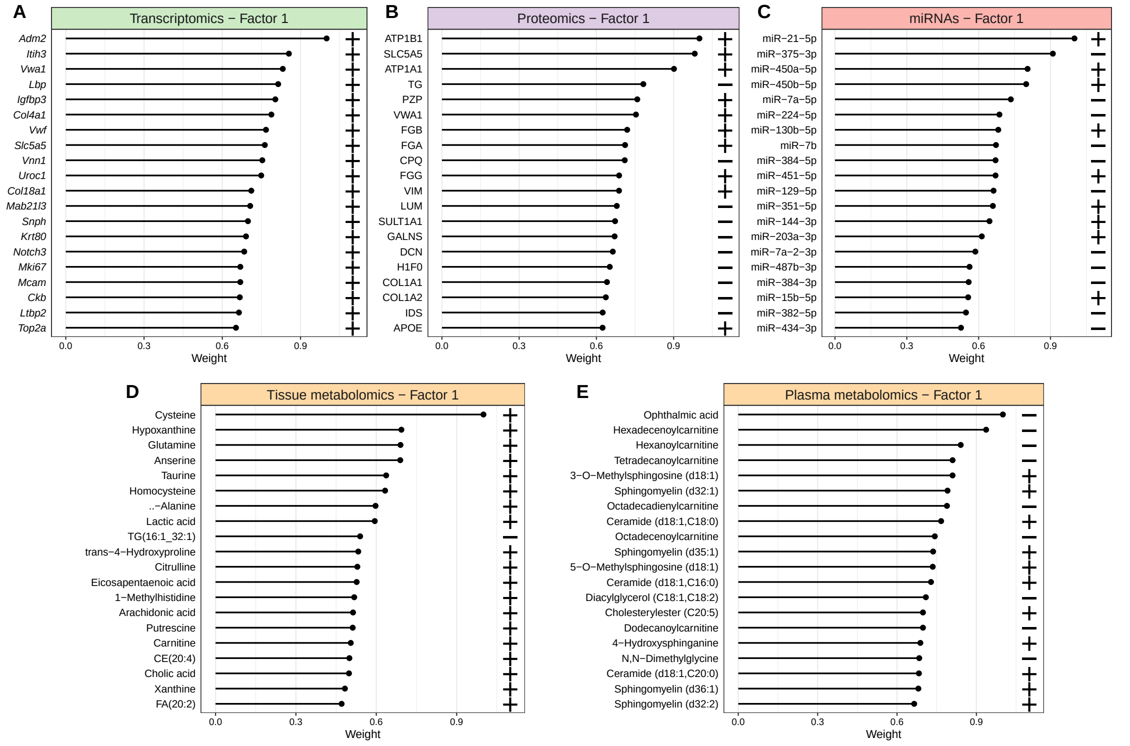

Fig. S8: Feature weights of omics layers in the thyroid PTU model. For each omics layer, the Top 20 features with the highest absolute feature weights in latent factor 1 are shown.

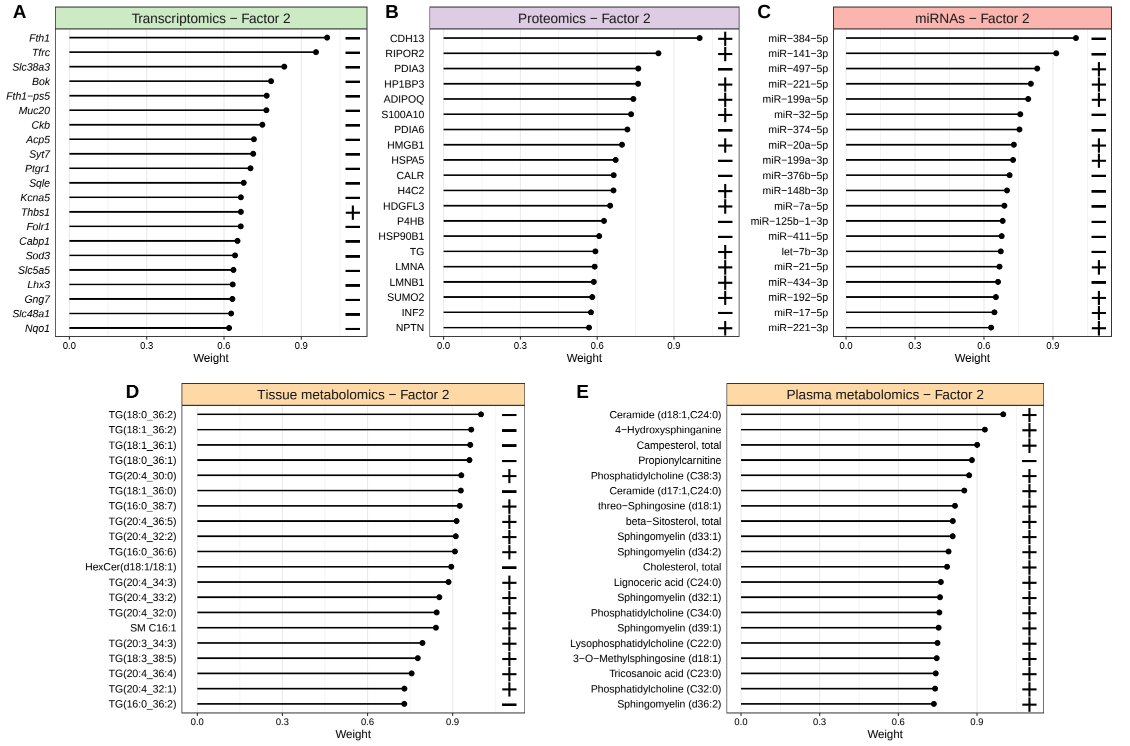

Fig. S9: Feature weights of omics layers in the thyroid PTU model. For each omics layer, the Top 20 features with the highest absolute feature weights in latent factor 2 are shown.

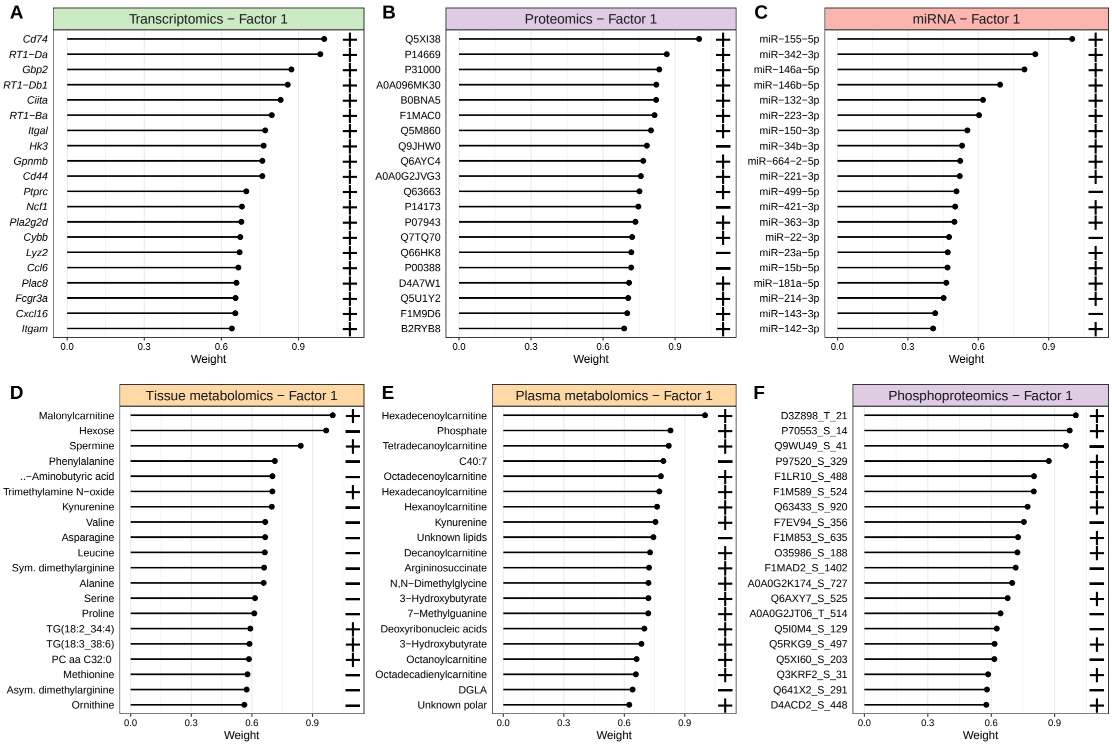

Fig. S10: Feature weights of omics layers in the liver Phenytoin model. For each omics layer, the Top 20 features with the highest absolute feature weights in latent factor 1 are shown.

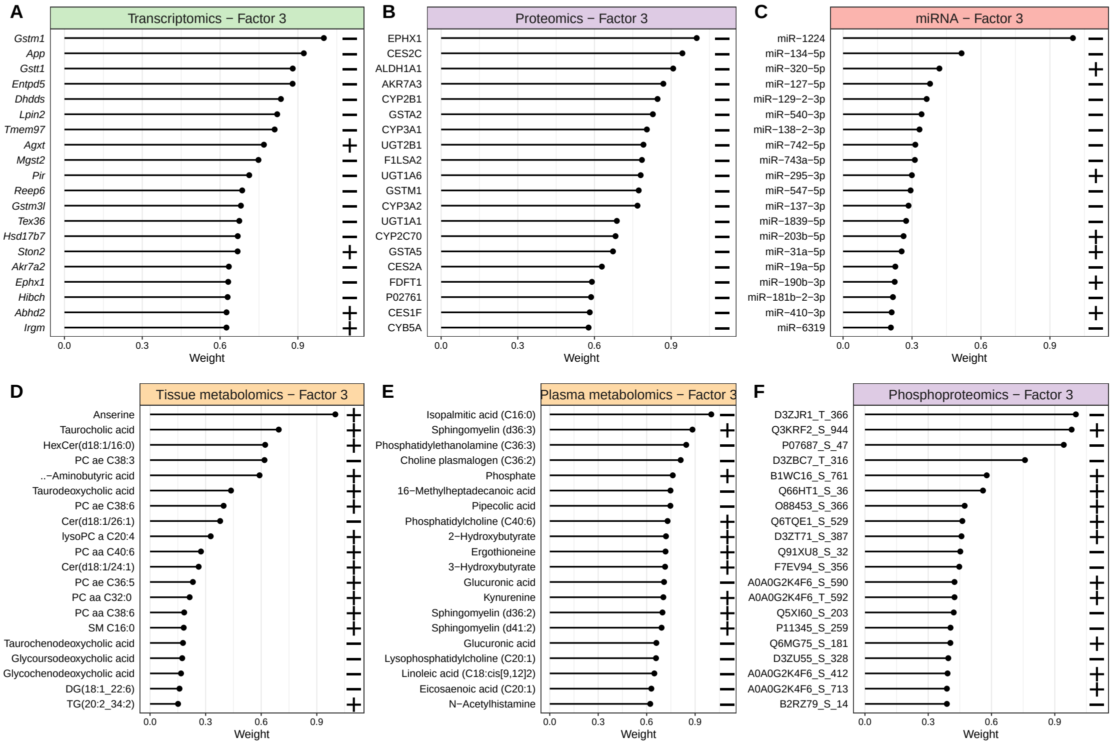

Fig. S11: Feature weights of omics layers in the liver Phenytoin model. For each omics layer, the Top 20 features with the highest absolute feature weights in latent factor 3 are shown.

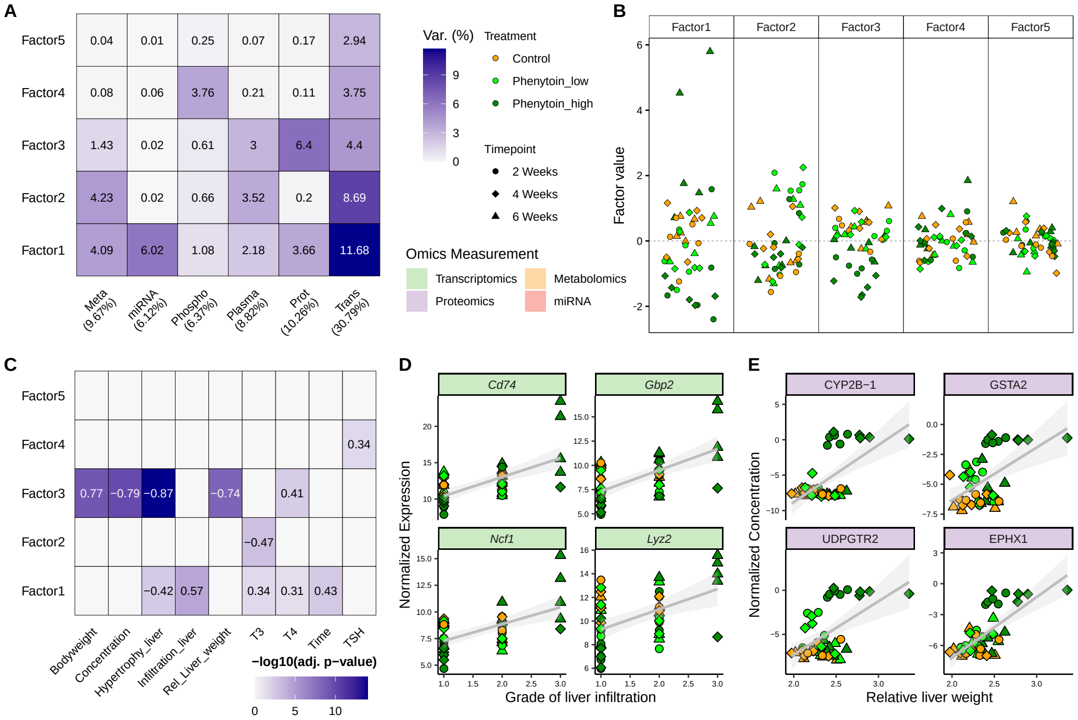

Fig. S12: Multi-omics data integration with the MEFISTO framework including clinical and histopathological parameters as covariates for the liver Phenytoin samples. (A) Variance captured across omics layers and latent factors. Factor 1 captures by far the most variance across all six omics layers. (B) Visualization of each factor capturing the global source of variability. Factor 1 indicates a high-dose Phenytoin response (dark green samples at the bottom) and two recovery samples with totally distant factor values. Factor 3 shows also separation of high-dose Phenytoin treated samples. (C) Heatmap showing the correlation of clinical and histopathological parameters with factor values. The Pearson correlation is written within the cell when the correlation was significant. The significance level is color coded. (D) Correlation of normalized expression values of transcriptomics features with high weights in latent factor 1 against grades of liver infiltrations. The severity of those infiltrations is measured in grades from 0 to 5, with 5 being the most severe. (E) Correlation of normalized concentrations of proteins with high weights in latent factor 3 against the relative liver weights.

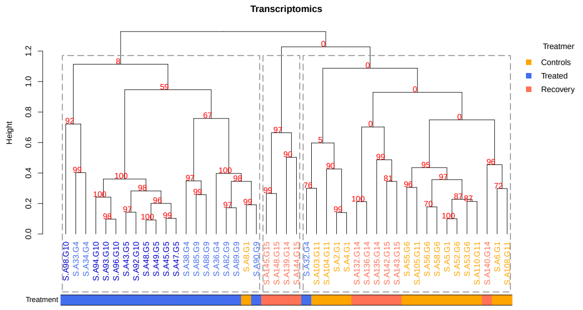

(a)

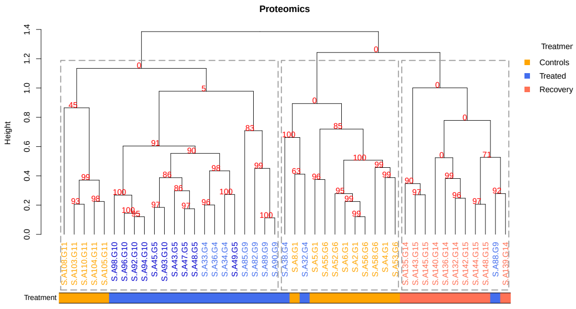

(b)

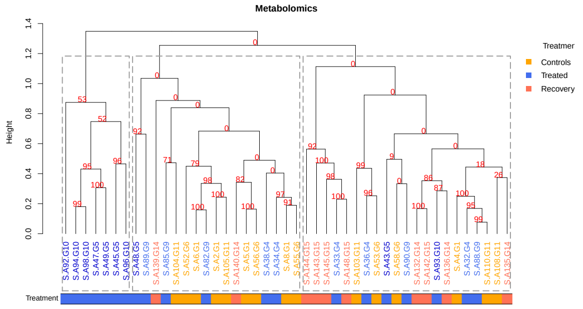

(c)

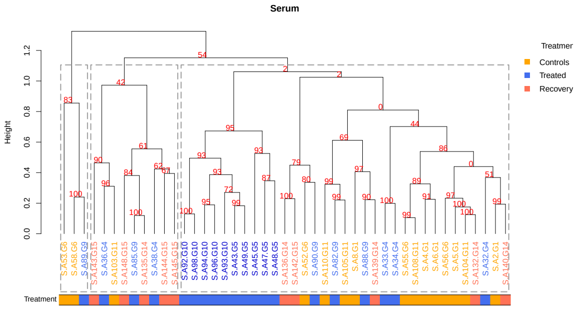

(d)

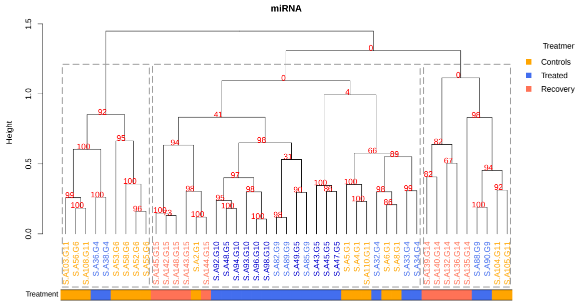

(e)

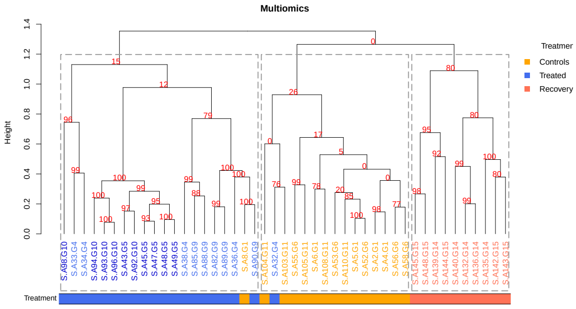

(f)

Fig. S13: Clustering of PTU thyroid samples. `MEFISTO`-derived factor weights of each sample were used to calculate their euclidean distance and an hierarchical clustering. Bootstrap support for a subtree is written in red at each respective node. Each sample is colored based on its treatment group, where orange, blue, and coral indicate controls, PTU treatments, and recovery samples, respectively. The time parameter is neglected in this clustering. Cluster dendrograms of the single omics models are shown in (A) - (G), while the multi-omics model is shown in (F).

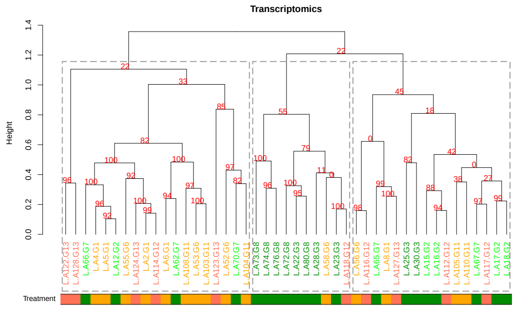

(a)

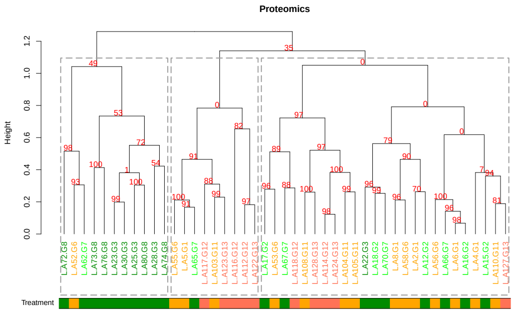

(b)

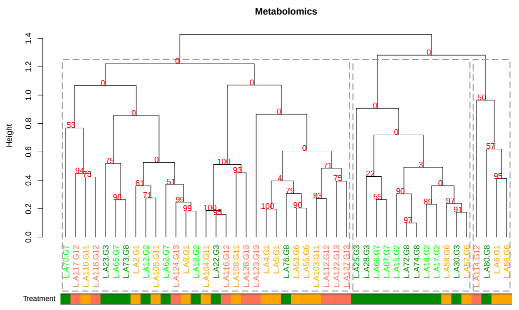

(c)

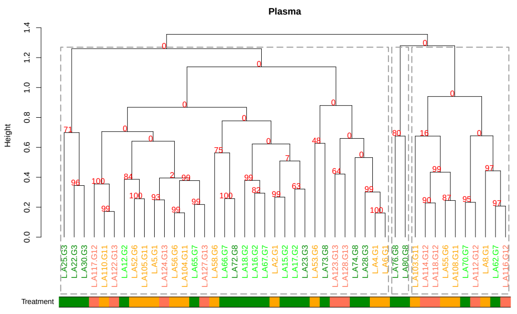

(d)

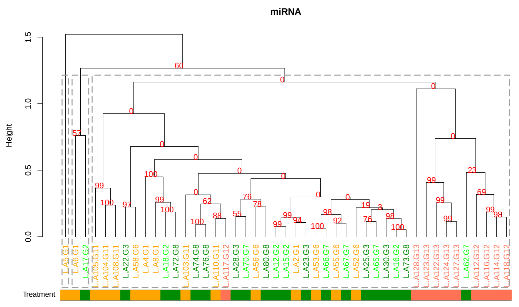

(e)

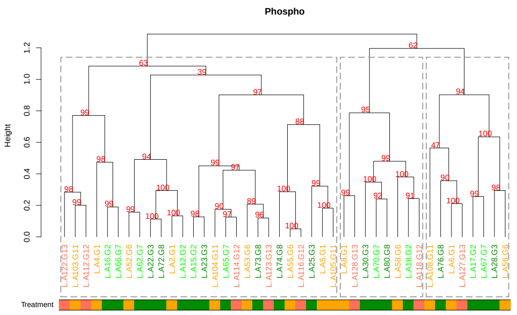

(f)

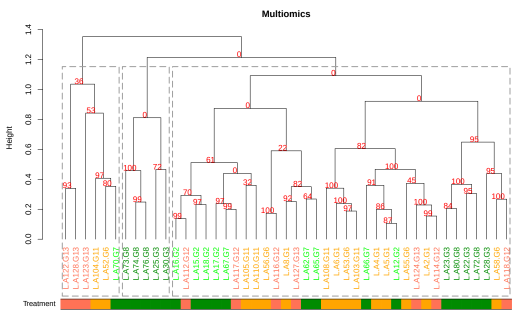

(g)

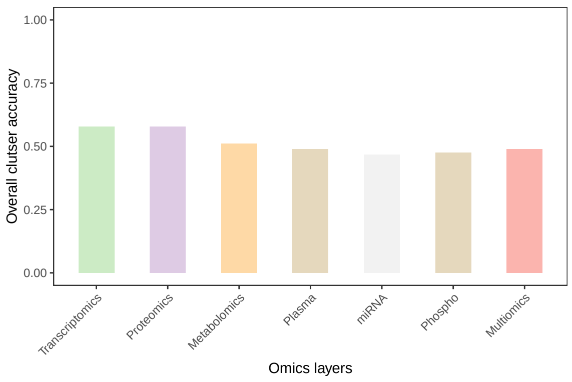

(h)

Fig. S14: Clustering of Phenytoin liver samples. `MEFISTO`-derived factor weights of each sample were used to calculate their euclidean distance and an hierarchical clustering. Bootstrap support for a subtree is written in red at each respective node. Each sample is colored based on its treatment group, where orange, green, and coral indicate controls, Phenytoin treatments, and recovery samples, respectively. The time parameter is neglected in this clustering. Cluster dendrograms of the single omics models are shown in (A) - (F), while the multi-omics model is shown in (G). Comparison of clustering accuracy of single-omics and multi-omics `MEFISTO` models is depicted in (H).

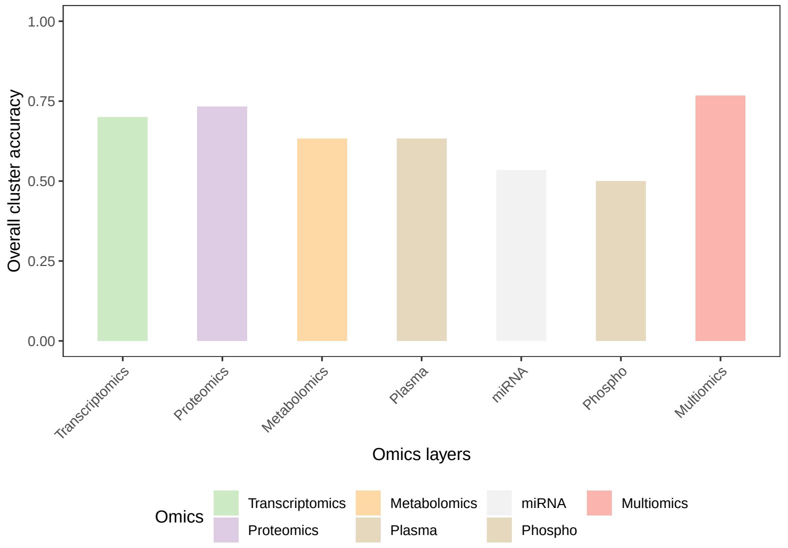

Fig. S15: Comparison of clustering accuracies of Phenytoin liver samples on a reduced set of samples. The approach is similar to the one presented in Figure S14. The low-dose Phenytoin treated samples have been left out prior to the model generation.

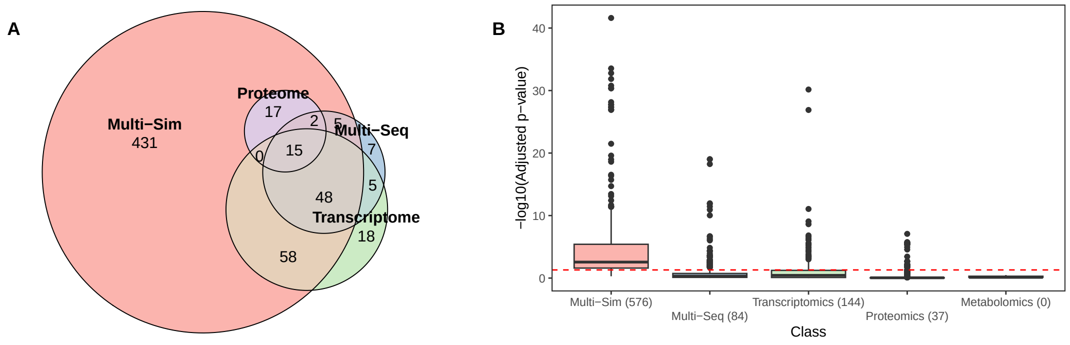

Fig. S16: Comparison of single- and multi-omics-based approaches for pathway enrichments in the liver Phenytoin model. All enrichments were calculated using `multiGSEA`. Single-omics and the sequential, log2 fold change-based multi-omics enrichment (Multi-Seq) used results of the previous differential expression analysis, while the simultaneous approach utilized the `MEFISTO`-derived factor weights as ranking metric (Multi-Sim). (A) Comparison of the number of enriched pathways between single-and multi-omics pathway enrichments. (B) Boxplot indicating the significance level of all pathways that have been significantly enriched with at least one enrichment approach (FDR < 0.05).

Table S2: Log2 fold changes of controls samples versus genes, proteins, and metabolites after PTU treatment in thyroid tissue. Metabolites are distinguished between thyroid tissue and plasma. Significant changes are indicated in bold. FDR smaller than 0.01 is marked with '*'; FDR smaller than 0.001 is marked with '**'.

		Ctrl vs low-dose PTU (log2FC)			Ctrl vs high-dose PTU (log2FC)		
Feature	Type	2 weeks	4 weeks	Recovery	2 weeks	4 weeks	Recovery
Cdo1	Gene	0.6613	0.489	0.1566	-0.9432	-0.3905	-0.5136
Tpo	Gene	0.355	**0.5803***	-0.1384	0.4196	**0.4747***	-0.3515
Tg	Gene	0.0715	0.2281	-0.0936	-0.0322	-0.2332	**-0.4639****
Gclc	Gene	-0.1518	0.529	-0.1727	-0.3312	-0.0122	-0.1787
Ctsb	Gene	0.2203	0.0983	-0.2269	-0.1294	**-0.3975****	**-0.5649****
LOC103691744	Gene	0.5304	0.9638	-0.0026	1.1688	0.44	-0.9285
Csad	Gene	0.1516	0.2381	-0.2465	0.1297	0.1253	**-0.3997****
Gclm	Gene	0.0141	0.1632	-0.0436	0.0697	0.2123	-0.124
Gss	Gene	0.1356	**0.3834***	-0.2976	0.0726	0.2447	**-0.5163****
Cbs	Gene	0.3464	**1.4869****	-0.3358	0.2451	**1.1163****	-0.3626
CTSB	Protein	-3.23	-2.89	-3.37	**-4.3****	**-3.85***	**-5.06****
TG	Protein	-2.57	-4.58	1.15	-3.11	**-5.18****	0.138
TPO	Protein	0.29	1.68	-0.997	0.58	0.89	-1.73
GCLC	Protein	0.1	-0.45	0.301	-0.55	-0.934	-0.146
GSS	Protein	0.0708	1.65	0.722	-0.85	0.121	0.655
GCLM	Protein	1.74	1.99	0.319	1.31	0.897	-0.812
CSAD	Protein	0.132	0.357	0.166	-0.323	0.355	0.16
Taurine	Metabolite (Tissue)	0.473	0.245	0.0857	**2.74****	**2.84****	1.29
Cysteine	Metabolite (Tissue)	1.28	0.251	0.404	**4.93****	**4.63****	1.15
Homocysteine	Metabolite (Tissue)	1.18	1.01	0.496	**3.19***	**3.66***	0.964
Glutamine	Metabolite (Tissue)	1.07	0.587	0.823	**3.31****	**3.13****	1.14
Methionine	Metabolite (Tissue)	-0.0974	-0.51	-0.282	1.02	0.837	0.0981
Serine	Metabolite (Tissue)	-0.108	-0.327	-0.112	1.44	1.39	0.305
Methionine	Metabolite (Plasma)	0.0373	0.235	0.0255	0.0683	0.0102	-0.198
Cysteine	Metabolite (Plasma)	-0.0161	-0.156	-0.118	-0.283	-0.298	-0.0308
Glutamine	Metabolite (Plasma)	-0.0256	0.0043	-0.0821	0.0477	-0.166	-0.145
Serine	Metabolite (Plasma)	0.175	0.324	0.104	-0.186	-0.0324	-0.0654
Taurine	Metabolite (Plasma)	0.283	-0.286	0.328	0.466	0.12	0.148
Homocysteine	Metabolite (Plasma)	-0.316	0.788	-0.244	-0.132	-0.593	-0.379
Taurine	Metabolite (Plasma)	0.262	0.0493	0.285	0.245	0.22	0.0267
Glutamine	Metabolite (Plasma)	-0.026	0.142	0.056	-0.222	-0.362	-0.115

Table S3: Log2 fold changes of controls samples versus genes, proteins, and metabolites after PTU treatment in liver tissue. Metabolites are distinguished between liver tissue and plasma. Significant changes are indicated in bold. FDR smaller than 0.01 is marked with '*'; FDR smaller than 0.001 is marked with '**'.

		Ctrl vs low-dose PTU (log2FC)			Ctrl vs high-dose PTU (log2FC)		
Feature	Type	2 weeks	4 weeks	Recovery	2 weeks	4 weeks	Recovery
Cdo1	Gene	-0.1344	-0.0416	-0.1076	**-0.8514****	-0.4412	-0.0965
Tpo	Gene	-0.3054	-4.1975	0.2061	1.0226	-4.086	-0.4082
Tg	Gene	-0.4886	-4.5969	0.0476	0.5918	-3.8966	-0.475
Gclc	Gene	0.3695	-0.0035	0.1363	-0.1988	-0.1798	0.2711
Ctsb	Gene	-0.1384	-0.2015	0.1126	-0.0585	0.1067	-0.0199
LOC103691744	Gene	-0.0041	-0.0131	0.1202	**0.9267****	0.4298	-0.3433
Csad	Gene	0.273	0.2843	0.2607	**-0.9077****	0.4713	0.4091
Gclm	Gene	0.389	0.1559	0.0651	-0.1142	-0.0445	0.2471
Gss	Gene	0.0232	0.1443	0.0668	-0.3537	-0.1419	0.078
Cbs	Gene	-0.4168	-0.0984	0.046	-0.1763	0.2477	-0.2165
GCLC	Protein	1.03	-0.871	0.165	0.758	-0.221	1.4
CDO	Protein	-1.98	-0.956	0.0033	-1.89	-0.915	-2.33
CBS	Protein	-0.439	0.0407	-0.393	0.718	-0.23	-2.03
GSS	Protein	0.868	0.314	0.442	0.33	0.332	1.59
GCLM	Protein	0.523	-0.411	-0.305	-0.888	-0.0682	0.746
CSAD	Protein	1.76	-0.427	1.3	-0.64	0.245	1.71
CTSB	Protein	0.117	0.555	-0.0657	0.914	0.537	-0.177
CTH	Protein	0.706	-0.436	0.495	3.56	4.1	1.2
Glutamine	Metabolite (tissue)	**1.15***	0.387	0.5	0.989	1.25	0.609
Methionine	Metabolite (tissue)	-0.109	-0.11	-0.28	-0.407	-0.629	-0.268
Serine	Metabolite (tissue)	-0.0782	-0.103	-0.0514	-0.662	-1.08	0.16
Homocysteine	Metabolite (tissue)	0.65	0.141	0.618	-0.165	0.542	0.597
Taurine	Metabolite (tissue)	0.205	0.313	-0.0755	0.569	0.632	-0.71
Cysteine	Metabolite (tissue)	1.62	0.136	0.0452	-0.0458	-0.641	1.65
Methionine	Metabolite (plasma)	0.0373	0.235	0.0255	0.0683	0.0102	-0.198
Cysteine	Metabolite (plasma)	-0.0161	-0.156	-0.118	-0.283	-0.298	-0.0308
Glutamine	Metabolite (plasma)	-0.0256	0.0043	-0.0821	0.0477	-0.166	-0.145
Serine	Metabolite (plasma)	0.175	0.324	0.104	-0.186	-0.0324	-0.0654
Taurine	Metabolite (plasma)	0.283	-0.286	0.328	0.466	0.12	0.148
Homocysteine	Metabolite (plasma)	-0.316	0.788	-0.244	-0.132	-0.593	-0.379
Taurine	Metabolite (plasma)	0.262	0.0493	0.285	0.245	0.22	0.0267
Glutamine	Metabolite (plasma)	-0.026	0.142	0.056	-0.222	-0.362	-0.115

Table S4: Log2 fold changes of genes, proteins, and miRNAs after PTU treatment in thyroid samples. The features illustrated here are examples to highlight the benefits of multi-omics data integration for identifying post-transcriptional regulation. For more details, see Section 4 in the main manuscript. Significant changes are indicated in bold.

		Low-dose PTU				High-dose PTU			
		2 Weeks		4 Weeks		2 Weeks		4 Weeks	
miRNA	Type	log2FC	P.adj	log2FC	P.adj	log2FC	P.adj	log2FC	P.adj
miR-21-5p	miRNA	0.198	0.477	0.851	**2.43e-07**	2.044	**3.25e-43**	2.615	**5.71e-67**
miR-224-5p	miRNA	-0.232	0.585	-1.463	**3.80e-08**	-1.353	**1.21e-07**	-2.069	**2.29e-15**
miR-7a-2-3p	miRNA	-0.018	1	-0.582	0.123	-1.389	**2.51e-04**	-2.456	**4.75e-09**
miR-199a-5p	miRNA	0.045	1	0.406	0.046	0.869	**7.46e-06**	1.284	**9.80e-12**
Dio1	gene	-0.371	0.015	-0.186	0.111	-1.22	**6.77e-10**	-2.236	**5.63e-32**
Snai1	gene	0.195	0.139	0.025	0.907	1.549	**6.25e-12**	1.235	**3.27e-08**
Cdh1	gene	0.086	0.281	0.142	0.068	0.128	0.108	0.123	0.124
CDH1	protein	0.865	0.23	1.25	0.044	-0.092	0.909	1.42	0.01
Basp1	gene	0.063	0.596	-0.003	0.979	1.51	**3.91e-06**	1.184	**3.12e-04**
BASP1	protein	-3.56	0.201	-2.15	0.403	-4.62	0.047	-4.74	0.03

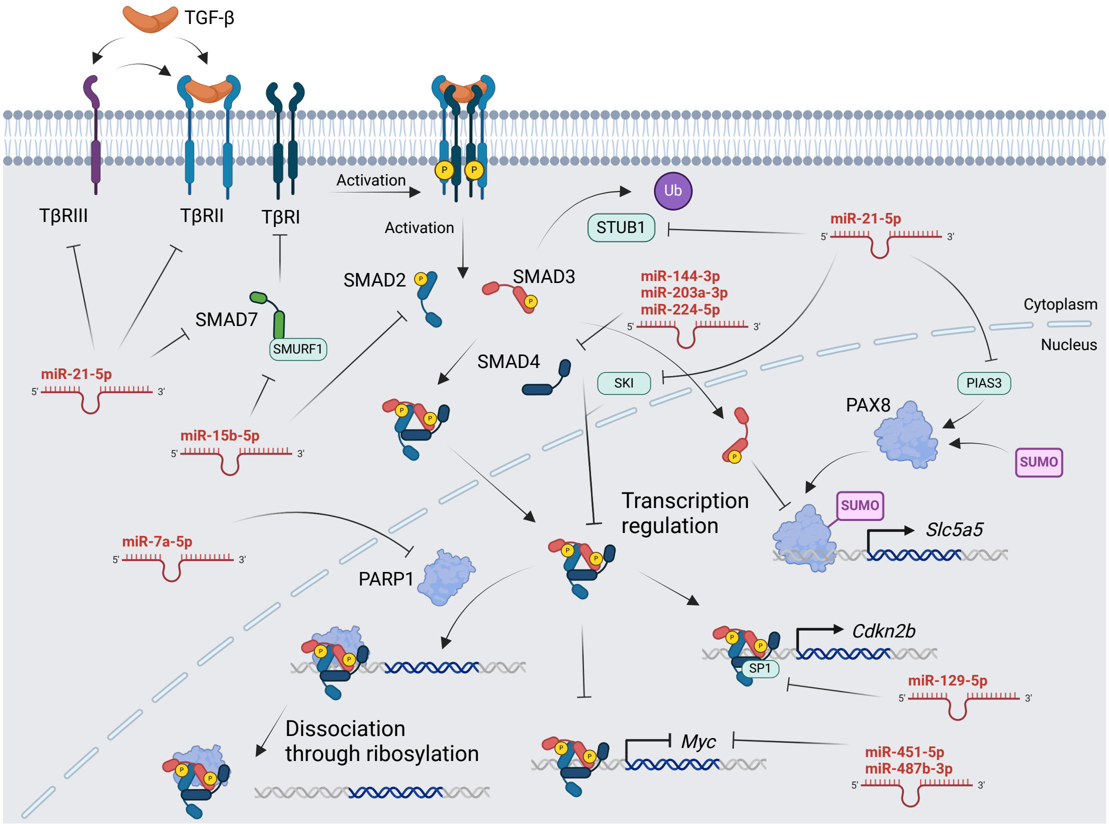

Fig. S17: Linking miRNAs with TGF-β signaling, SMAD-signaling and SUMOylation to regulate thyroid functioning. MiRNAs shown in this pathway illustration were found to have high feature weights in LF1 in the thryroid PTU model. Created with BioRender.com.

S4 Linking miRNA pathway enrichment with multi-omics analysis

As detailed in the main manuscript, we utilized the Top 20 miRNAs with the highest feature weights in latent factor 1 in the thyroid PTU multi-omics model and conducted a miRNA-based pathway enrichment using `miRPATH-v4.0` (Tastsoglou et al., 2023). The miRNAs used can be seen in Supplementary Figure S8(C). Detailed results of this enrichment are available in the corresponding Supplementary file in our git repository[1].

Significantly enriched pathways in the miRNA layer include TGF-β signaling, SUMOylation, and SMAD-related pathways, which regulate thyroid follicular cell differentiation, growth, and function. Thyroid-stimulating hormone (TSH) and transforming growth factor (TGF-β) are key signals in these processes (López-Márquez et al., 2022), acting through cAMP/CREB and SMAD pathways to regulate thyroid differentiation markers such as PAX8 (Medina et al., 2000), thyroglobulin (TG) (Christophe et al., 1989), thyroperoxidase (TPO) (Damante et al., 1989), and the Na^+/I^- symporter (NIS) (Costamagna et al., 2004). A graphical overview of the interplay of genes/proteins with high feature weight miRNAs is shown in Figure S17.

Tgf-β exists in three isoforms - *Tgf-β1*, *Tgf-β2*, and *Tgf-β3*. *Tgf-β* expression is upregulated in TSH-induced thyroid hyperplasia in rats (Logan et al., 1994). We also detected significant upregulation of this ligand, as shown in Supplementary Tables S7 and S8. *Tgf-β1* is a target of miR-144-3p, which is upregulated and shows high feature weights in LF1 (see Supplementary

[1] https://codebase.helmholtz.cloud/department-computational-biology/xometox/process_integrate_omics_data/-/blob/master/miRNA_pathway_enrichment/01_thyroid_miRNA_pathway_enrichment.Rmd

Table S5). A summary of these miRNAs and their target genes involved in thyroid regulatory mechanisms is in Supplementary Table S6.

TGF-β signaling is mediated by three receptor types: TGF-βRI, TGF-βRII, and TGF-βRIII. TGF-β binds to TGF-βRIII or directly to TGF-βRII, which recruits TGF-βRI to form a complex that activates SMAD2 and SMAD3 transcription factors (Deng et al., 2024). All three receptors were upregulated at the transcriptome level, with *Smad2* slightly upregulated (significant change after two weeks) and *Smad3* upregulated after two and four weeks. Besides other targets, miR-21-5p targets *Tgf-βrII* and *Tgf-βrIII* receptors in human (Kim et al., 2009; Yu et al., 2012). Its rat homolog was significantly upregulated after PTU treatment after two and four weeks of high-dose PTU treatment. *Smad2* is targeted by miR-15b-5p, which was also significantly upregulated.

SMAD2/3 associate with its partner protein SMAD4 to form a heterotrimer accumulating in the nucleus to regulate target genes expression. For example, it negatively affects the MYC transcription factors (Chen et al., 2002). The upregulation of MYC, however, has been detected in thyroid cancer to promote cell proliferation, reduce cell apoptosis, and enhance cell malignant transformation to become tumorigenic (Deng et al., 2024). Therefore, TGF-β can provide additional protection against tumorigenesis. We found *Myc* to be slighly up-regulated though after four weeks of PTU treatment. *Myc* is furthermore targeted by two important miRNAs in LF1: miR-451-5p and miR-487b-3p with the first one being up-regulated and the latter one being down-regulated. After binding with SP1, the SMAD heterotrimer positively effects the expression of *Cdkn2b* (Feng et al., 2000), a cell-cycle regulator that was regularly found to have promotive effects on thyroid cancer (Wu et al., 2022). In this case TGF-β signaling promotes the growth and metastasis of thyroid cancer types. *Sp1* showed no regulation of expression, while *Cdkn2b* was significantly upregulated. *Sp1* is furthermore a known target of miR-129-5p, which was found to be significantly down-regulated.

However, there are several other processes besides post-transcriptional regulation that regulate the TGF-β induced signaling. For example, the formation of both TGF-β receptors I and II is inhibited by SMAD7 in combination with SMURF1 (Nakao et al., 1997; Yan et al., 2009). Both were found to be significantly upregulated on transcriptome level. Furthermore, *Smad7* and *Smurf1* are also targets of the previously mentioned miR-21-5p and miR-15b-5p, respectively. Another example is PARP1, which is a key player in controling strength and duration of SMAD-mediated transcription (Lönn et al., 2010). It dissociates SMAD complexes by ADP-ribosylation of SMAD3 and 4 attenuating SMAD-specific gene responses and the TGF-β-induced epithelial-mesenchymal transition. *Parp1* is a target of miR-7a-5p which was significantly downregulated during high-dose PTU treatment, even after a 2 weeks recovery. However, its transcript and protein level were not found to be differentially altered. SKI is able to locate certain repressors to the SMAD2/3:SMAD4 complex, thus inhibiting SMAD-mediated transcription and supressing TGF-β signaling (Sun et al., 1999). *Ski* is also a target of miR-21-5p but showed no sign of differential expression. *Smad4* is a validated target of miR-144-3p, miR-203a-3p (both were up-regulated), and miR-224-5p (down-regulated) and was found to be slightly but significantly down-regulated after two weeks high-dose PTU treatment.

Furthermore, SMAD3 reduces the expression of *Slc5a5* through its physical interaction with the transcription factor PAX8, which in turn diminishes PAX8 binding to the DNA sequence involved in the regulation of *Slc5a5* (Mincione et al., 2011). PAX8 is involved in the morphogenesis of the thyroid gland and in the maintenance of the differentiated thyroid phenotype since it is the main factor controlling *Slc5a5*, *Tg*, and *Tpo* transcription (Costamagna et al., 2004). Sumoylation of PAX8 is required for protein stability, and hence indirectly affecting the transcriptional activity (de Cristofaro et al., 2009). Although we found significant expression changes for several miRNAs that target genes that are involved in the sumoylation process, such as miR-21-5p targeting *Pias3*, we found no significant change on transcrtipome levels of those genes, pointing towards unaltered sumoylation of PAX8. The basal levels of SMAD3 are regulated by STUB1 which promotes the ubiquitin-mediated degradation Xin et al. (2005). *Stub1* is neither regulated on transcriptome level nor on proteome level although it is a known target of the up-regulated miR-21-5p Battaglia et al. (2019).

Table S5: Results of the differential expression analysis between controls vs low/high-dose treated samples for high weight miRNAs in LF1 of the thyroid PTU model. The miRNAs that are shown in the table are inbolved in TGF-β-signaling, SMAD-related signaling, or SUMOylation.

	Low-dose PTU						High-dose PTU					
	2 Weeks		4 Weeks		6 Weeks		2 Weeks		4 Weeks		6 Weeks	
miRNA	l2FC	P.adj	l2FC	P.adj	l2FC	P.adj	l2FC	P.adj	l2FC	P.adj	l2FC	P.adj
miR-21-5p	0.198	0.477	0.851	**2.43e-07**	-0.246	0.309	2.044	**3.25e-43**	2.615	**5.71e-67**	0.859	**1.94e-07**
miR-224-5p	-0.232	0.585	-1.463	**3.80e-08**	-0.281	0.491	-1.353	**1.21e-07**	-2.069	**2.29e-15**	-0.797	**2.98e-03**
miR-451-5p	0.283	0.499	0.648	0.132	0.085	1	0.976	0.039	1.44	**4.18e-03**	-0.186	0.866
miR-129-5p	-0.145	0.911	-1.113	**8.74e-04**	-0.709	0.033	-1.344	**1.98e-05**	-2.264	**2.65e-12**	-0.815	0.011
miR-144-3p	0.366	0.291	1.427	**2.28e-03**	0.241	0.743	1.228	**6.44e-03**	2.265	**8.37e-06**	0.079	1
miR-203a-3p	0.651	**6.46e-03**	1.279	**3.09e-08**	0.032	1	1.305	**2.87e-09**	1.771	**1.30e-15**	-0.077	0.976
miR-7a-2-3p	-0.018	1	-0.582	0.123	-0.278	0.641	-1.389	**2.51e-04**	-2.456	**4.75e-09**	-1.487	**4.25e-05**
miR-487b-3p	-0.196	0.757	-1.267	**3.15e-04**	-0.109	0.968	-1.118	**9.26e-04**	-1.639	**3.30e-06**	-0.504	0.181
miR-15b-5p	0.759	**2.97e-07**	0.445	**2.84e-03**	-0.14	0.629	1.311	**1.35e-22**	1.045	**4.03e-14**	0.716	**1.05e-06**

Table S6: Target genes that are affected by miRNAs that showed high weights in the LF1 of the thyroid PTU model. The genes shown here are involved in TGF-β signaling, SMAD-signaling or SUMOylation pathways.

Gene	miRNA
Tgfβ1	miR-144-3p
TgfβrII	miR-21-5p
TgfβrIII	miR-21-5p
Smad2	miR-15b-5p
Smad4	miR-144-3p, miR-203a-3p, miR-224-5p
Smad7	miR-21-5p, miR-15b-5p
Parp1	miR-7a-5p
Ski	miR-21-5p
Smurf	miR-15b-5p
Myc	miR-451-5p, miR-487b-3p
Pias3	miR-21-5p
Sp1	miR-129-5p
Stub1	miR-21-5p

Table S7: Results of the differential expression analysis between controls vs low/high-dose treated samples for genes that are involved in TGF-β-signaling, SMAD-related signaling, or SUMOylation pathways.

	Low-dose PTU						High-dose PTU					
	2 Weeks		4 Weeks		6 Weeks		2 Weeks		6 Weeks		6 Weeks	
Gene	l2FC	P.adj	l2FC	P.adj	l2FC	P.adj	l2FC	P.adj	l2FC	P.adj	l2FC	P.adj
Smurf1	0.246	0.028	0.064	0.518	0.029	0.813	0.561	**1.07e-06**	0.456	**4.16e-05**	0.051	0.932
Tgfbr3	-0.081	0.339	-0.326	**6.51e-04**	0.097	0.271	-0.467	**4.17e-06**	-0.436	**1.22e-05**	0.197	0.048
Parp1	-0.154	0.074	0.005	0.897	0.018	0.939	-0.099	0.312	-0.021	0.936	0.031	0.677
Myc	0.085	0.372	0.712	**7.24e-03**	0.022	0.812	0.341	0.069	0.813	**2.81e-03**	0.148	0.225
Tpo	0.193	0.078	0.472	**1.40e-03**	-0.042	0.689	0.317	0.015	0.381	**4.54e-03**	-0.222	0.062
Tg	0.037	0.634	0.143	0.134	-0.039	0.718	-0.022	0.789	-0.177	0.089	-0.389	**5.57e-04**
Cdkn2b	1.429	**2.40e-06**	0.671	0.011	0.218	0.07	1.723	**3.98e-10**	1.757	**3.13e-10**	0.812	**1.91e-03**
Tgfbr1	0.016	0.833	-0.035	0.664	-0.024	0.851	0.265	**3.67e-04**	0.278	**1.27e-04**	0.038	0.825
Smad3	0.08	0.351	0.124	0.173	-0.029	0.82	0.471	**6.04e-06**	0.426	**2.82e-05**	0.208	0.035
Tgfb3	0.47	**6.31e-03**	0.131	0.237	0.049	0.589	1.825	**6.75e-19**	1.592	**5.80e-15**	0.97	**4.92e-06**
Tgfbr2	0.038	0.581	-0.018	0.908	0.086	0.343	0.469	**6.08e-04**	0.331	**0.009**	0.474	**8.25e-04**
Sp1	-0.084	0.14	-0.082	0.166	0.138	0.026	-0.06	0.293	-0.096	0.074	0.21	**1.03e-04**
Sumo1	-0.063	0.446	0.008	0.966	-0.013	0.965	-0.115	0.167	-0.066	0.516	-0.161	0.043
Smad2	0.064	0.418	-0.03	0.796	0.11	0.167	0.202	**8.54e-03**	0.135	0.068	0.173	0.027
Smad7	0.406	0.01	0.776	**6.98e-05**	-0.025	0.832	0.532	**1.03e-03**	1.057	**3.49e-09**	-0.018	1
Slc5a5	1.328	**8.21e-05**	1.172	**3.65e-04**	-0.638	0.019	2.621	**2.15e-17**	2.436	**2.01e-15**	-1.305	**7.38e-06**
Stub1	-0.09	0.188	-0.012	0.898	-0.067	0.458	-0.045	0.576	-0.046	0.642	-0.127	0.09
Tgfb1	0.054	0.68	0.069	0.541	0.023	0.807	1.433	**4.20e-16**	1.485	**2.02e-17**	0.746	**6.51e-05**
Pias3	0.024	0.791	0.014	0.905	-0.105	0.24	-0.042	0.769	-0.058	0.505	-0.135	0.183
Pax8	-0.043	0.606	0.06	0.557	-0.019	0.889	-0.051	0.652	-0.114	0.405	0.054	0.592
Smad4	-0.118	0.05	-0.206	**1.57e-04**	0.061	0.414	-0.172	**1.49e-03**	-0.101	0.076	0.12	0.035
Ski	0.09	0.254	0.07	0.467	-0.031	0.768	0.256	**1.16e-03**	0.183	0.018	-0.027	0.809

In conclusion, the interplay of miRNAs with genes and proteins involved in pathways in thyroid differentiation and functioning reveals a complex molecular network of signals and regulatory mechanisms. The significant changes in miRNA and gene expression highlights the intricate regulation of thyroid function in response to PTU treatment. However, a better coverage of the proteomics layer, especially for those proteins that are miRNA targets, would have sufficiently facilitate the generation of hypothesis how miRNA regulation effects those regulatory processes.

References

Battaglia C, Venturin M, Sojic A, Jesuthasan N, Orro A, Spinelli R, Musicco M, De Bellis G, Adorni F (2019) Candidate genes and mirnas linked to the inverse relationship between cancer and alzheimer's disease: Insights from data mining and enrichment analysis. Front Genet 10:846, DOI 10.3389/fgene.2019.00846

Table S8: Results of the differential expression analysis between controls vs low/high-dose treated samples for proteins that are involved in TGF-β-signaling, SMAD-related signaling, or SUMOylation pathways.

	Low-dose PTU						High-dose PTU					
	2 Weeks		4 Weeks		6 Weeks		2 Weeks		6 Weeks		6 Weeks	
Protein	l2FC	P.adj	l2FC	P.adj	l2FC	P.adj	l2FC	P.adj	l2FC	P.adj	l2FC	P.adj
SLC5A5	3.85	**1.74e-03**	2.88	0.041	0.345	0.992	5.4	**4.89e-06**	5.31	**1.70e-05**	0.334	0.955
STUB1	-0.791	0.985	-0.482	0.931	-0.757	0.987	-0.886	0.947	0.245	0.918	-0.861	0.914
TPO	0.988	0.989	2.65	0.751	0.87	0.992	1.88	0.937	3.31	0.632	0.19	0.969
PARP1	0.123	0.993	-2.17	0.645	-0.26	0.994	0.35	0.97	-1.36	0.85	0.76	0.941
SUMO1	0.369	0.992	0.152	0.952	-1.41	0.982	-0.123	0.974	-0.35	0.917	0.038	0.971

Chen CR, Kang Y, Siegel PM, Massagué J (2002) E2f4/5 and p107 as smad cofactors linking the tgfbeta receptor to c-myc repression. Cell 110(1):19–32, DOI 10.1016/s0092-8674(02)00801-2

Christophe D, Gérard C, Juvenal G, Bacolla A, Teugels E, Ledent C, Christophe-Hobertus C, Dumont JE, Vassart G (1989) Identification of a camp-responsive region in thyroglobulin gene promoter. Mol Cell Endocrinol 64(1):5–18, DOI 10.1016/0303-7207(89)90060-9

Costamagna E, García B, Santisteban P (2004) The functional interaction between the paired domain transcription factor pax8 and smad3 is involved in transforming growth factor-beta repression of the sodium/iodide symporter gene. J Biol Chem 279(5):3439–46, DOI 10.1074/jbc.M307138200

de Cristofaro T, Mascia A, Pappalardo A, D'Andrea B, Nitsch L, Zannini M (2009) Pax8 protein stability is controlled by sumoylation. J Mol Endocrinol 42(1):35–46, DOI 10.1677/JME-08-0100

Damante G, Chazenbalk G, Russo D, Rapoport B, Foti D, Filetti S (1989) Thyrotropin regulation of thyroid peroxidase messenger ribonucleic acid levels in cultured rat thyroid cells: evidence for the involvement of a nontranscriptional mechanism. Endocrinology 124(6):2889–94, DOI 10.1210/endo-124-6-2889

Deng Z, Fan T, Xiao C, Tian H, Zheng Y, Li C, He J (2024) TGF-beta signaling in health, disease, and therapeutics. Signal Transduct Target Ther 9(1):61, DOI 10.1038/s41392-024-01764-w

Feng XH, Lin X, Derynck R (2000) Smad2, smad3 and smad4 cooperate with sp1 to induce p15(ink4b) transcription in response to tgf-beta. EMBO J 19(19):5178–93, DOI 10.1093/emboj/19.19.5178

Großkopf H, Walter K, Karkossa I, von Bergen M, Schubert K (2021) Non-genomic ahr-signaling modulates the immune response in endotoxin-activated macrophages after activation by the environmental stressor bap. Front Immunol 12:620270, DOI 10.3389/fimmu.2021.620270

Kim YJ, Hwang SJ, Bae YC, Jung JS (2009) Mir-21 regulates adipogenic differentiation through the modulation of tgf-beta signaling in mesenchymal stem cells derived from human adipose tissue. Stem Cells 27(12):3093–102, DOI 10.1002/stem.235

Logan A, Smith C, Becks GP, Gonzalez AM, Phillips ID, Hill DJ (1994) Enhanced expression of transforming growth factor-beta 1 during thyroid hyperplasia in rats. J Endocrinol 141(1):45–57, DOI 10.1677/joe.0.1410045

López-Márquez A, Carrasco-López C, Martínez-Cano A, Lemoine P, Pierreux CE, Santisteban P (2022) Sox9 is involved in the thyroid differentiation program and is regulated by crosstalk between tsh, tgf-beta and thyroid transcription factors. Sci Rep 12(1):2144, DOI 10.1038/s41598-022-06004-1

Lönn P, van der Heide LP, Dahl M, Hellman U, Heldin CH, Moustakas A (2010) Parp-1 attenuates smad-mediated transcription. Mol Cell 40(4):521–32, DOI 10.1016/j.molcel.2010.10.029

Medina DL, Suzuki K, Pietrarelli M, Okajima F, Kohn LD, Santisteban P (2000) Role of insulin and serum on thyrotropin regulation of thyroid transcription factor-1 and pax-8 genes expression in frtl-5 thyroid cells. Thyroid 10(4):295–303, DOI 10.1089/thy.2000.10.295

Mincione G, Di Marcantonio MC, Tarantelli C, D'Inzeo S, Nicolussi A, Nardi F, Donini CF, Coppa A (2011) Egf and tgf-1 effects on thyroid function. J Thyroid Res 2011:431718, DOI 10.4061/2011/431718

Nakao A, Afrakhte M, Morén A, Nakayama T, Christian JL, Heuchel R, Itoh S, Kawabata M, Heldin NE, Heldin CH, ten Dijke P (1997) Identification of smad7, a tgfbeta-inducible antagonist of tgf-beta signalling. Nature 389(6651):631–5, DOI 10.1038/39369

Roessner U, Wagner C, Kopka J, Trethewey RN, Willmitzer L (2000) Technical advance: simultaneous analysis of metabolites in potato tuber by gas chromatography-mass spectrometry. Plant J 23(1):131–42, DOI 10.1046/j.1365-313x.2000.00774.x

Sun Y, Liu X, Eaton EN, Lane WS, Lodish HF, Weinberg RA (1999) Interaction of the ski oncoprotein with smad3 regulates tgf-beta signaling. Mol Cell 4(4):499–509, DOI 10.1016/s1097-2765(00)80201-4

Tastsoglou S, Skoufos G, Miliotis M, Karagkouni D, Koutsoukos I, Karavangeli A, Kardaras FS, Hatzigeorgiou AG (2023) Diana-mirpath v4.0: expanding target-based mirna functional analysis in cell-type and tissue contexts. Nucleic Acids Res 51(W1):W154–W159, DOI 10.1093/nar/gkad431

Walk TB, Looser R, Bethan B, Herold MM, Kamlage B, Schmitz O, Wiemer JC, Prokoudine A, van Ravenzwaay B, Mellert W (2011) System and method for analyzing a sample using chromatography coupled mass spectrometry. Google Patents

Wu Q, He Y, Liu X, Luo F, Jiang Y, Xiang M, Zhao R (2022) Cancer stem cell-like cells-derived exosomal cdkn2b-as1 stabilizes cdkn2b to promote the growth and metastasis of thyroid cancer via tgf-1/smad2/3 signaling. Exp Cell Res 419(1):113268, DOI 10.1016/j.yexcr.2022.113268

Xin H, Xu X, Li L, Ning H, Rong Y, Shang Y, Wang Y, Fu XY, Chang Z (2005) Chip controls the sensitivity of transforming growth factor-beta signaling by modulating the basal level of smad3 through ubiquitin-mediated degradation. J Biol Chem 280(21):20842–50, DOI 10.1074/jbc.M412275200

Yamada H, Yamahara A, Yasuda S, Abe M, Oguri K, Fukushima S, Ikeda-Wada S (2002) Dansyl chloride derivatization of methamphetamine: a method with advantages for screening and analysis of methamphetamine in urine. J Anal Toxicol 26(1):17–22, DOI 10.1093/jat/26.1.17

Yan X, Liu Z, Chen Y (2009) Regulation of tgf-beta signaling by smad7. Acta Biochim Biophys Sin (Shanghai) 41(4):263–72, DOI 10.1093/abbs/gmp018

Yu Y, Kanwar SS, Patel BB, Oh PS, Nautiyal J, Sarkar FH, Majumdar AP (2012) MicroRNA-21 induces stemness by downregulating transforming growth factor beta receptor 2 (TGFBR2) in colon cancer cells. Carcinogenesis 33(1):68–76, DOI 10.1093/carcin/bgr246

Zhang F, Rick DL, Kan LH, Perala AW, Geter DR, LeBaron MJ, Bartels MJ (2011) Simultaneous quantitation of testosterone and estradiol in human cell line (h295r) by liquid chromatography/positive atmospheric pressure photoionization tandem mass spectrometry. Rapid Commun Mass Spectrom 25(20):3123–30, DOI 10.1002/rcm.5208
